# Genetic markers associated to arbuscular mycorrhizal colonization in durum wheat

**DOI:** 10.1038/s41598-018-29020-6

**Published:** 2018-07-13

**Authors:** Pasquale De Vita, Luciano Avio, Cristiana Sbrana, Giovanni Laidò, Daniela Marone, Anna M. Mastrangelo, Luigi Cattivelli, Manuela Giovannetti

**Affiliations:** 1 Consiglio per la ricerca in agricoltura e l’analisi dell’economia agraria, Centro di Ricerca Cerealicoltura e Colture Industriali, S.S. 673 km 25 + 200, 71121 Foggia, Italy; 2 Dipartimento di Scienze Agrarie, Alimentari e Agro-Ambientali, Università di Pisa, Via del Borghetto 80, 56124 Pisa, Italy; 3 Istituto di Biologia e Biotecnologia Agraria CNR, Pisa, Italy; 4 Consiglio per la ricerca in agricoltura e l’analisi dell’economia agraria, Centro di Ricerca Genomica e Bioinformatica, Via San Protaso 302, 29017 Fiorenzuola d’Arda, (PC) Italy; 5 Present Address: Consiglio per la ricerca in agricoltura e l’analisi dell’economia agraria, Centro di Ricerca Cerealicoltura e Colture Industriali, Via Stezzano 24, 24126 Bergamo, Italy

## Abstract

In this work we investigated the variability and the genetic basis of susceptibility to arbuscular mycorrhizal (AM) colonization of wheat roots. The mycorrhizal status of wild, domesticated and cultivated tetraploid wheat accessions, inoculated with the AM species *Funneliformis mosseae*, was evaluated. In addition, to detect genetic markers in linkage with chromosome regions involved in AM root colonization, a genome wide association analysis was carried out on 108 durum wheat varieties and two AM fungal species (*F*. *mosseae* and *Rhizoglomus irregulare*). Our findings showed that a century of breeding on durum wheat and the introgression of *Reduced height* (*Rht*) genes associated with increased grain yields did not select against AM symbiosis in durum wheat. Seven putative Quantitative Trait Loci (QTLs) linked with durum wheat mycorrhizal susceptibility in both experiments, located on chromosomes 1A, 2B, 5A, 6A, 7A and 7B, were detected. The individual QTL effects (*r*^2^) ranged from 7 to 16%, suggesting a genetic basis for this trait. Marker functional analysis identified predicted proteins with potential roles in host-parasite interactions, degradation of cellular proteins, homeostasis regulation, plant growth and disease/defence. The results of this work emphasize the potential for further enhancement of root colonization exploiting the genetic variability present in wheat.

## Introduction

Arbuscular mycorrhizal fungi (AMF) establish mutualistic symbiotic associations with the roots of most land plants^1^ and provide many different agroecosystem services, such as an efficient use of fertilizers and soil nutrients, protection against biotic and abiotic stresses, increased N_2_ fixation in legume crops and improved soil aggregation^2^. They enhance plant nutrition by means of extensive mycelial networks which spread from colonized roots and efficiently absorb and translocate mineral nutrients, such as phosphorus (P) and nitrogen (N) from the soil to the host plants^3,4^. Host plant susceptibility to AMF - assessed by colonized root length measurements - is highly variable and depends on many factors, related to symbiont identity and its inoculum potential, environment (soil fertility, temperature and moisture) and plant genotype, including root traits^5–7^. The role of colonization rate on crop yield is still unresolved^8–11^, although many data corroborate its positive effect on plant growth^12,13^.

Wheat (*Triticum* spp.) is one of the most important food crops worldwide, whose wild relatives were domesticated by humans dating back more than 10,000 years^14^. The average yield of wheat has increased more than 3 times since 1900, mainly as a result of genetic improvement by breeding and of intensive management practices^15^. Some studies suggested that modern high-yielding genotypes, selected for the optimal performance in high fertility soils, may have lost the capacity to respond to AMF as a result of their ability to uptake phosphate and other soil mineral nutrients without the aid of their symbionts, compared to old varieties^16–18^. Although the breeding of crop genotypes able to benefit from AMF could be greatly enhanced by information on susceptibility of cultivated varieties to mycorrhizal colonization, scanty information is available on the influence of host genotype on the establishment of mycorrhizal symbiosis, and in particular on the putative loss of susceptibility of modern vs. old varieties to AMF^13,16,17,19,20^. Differences in plant susceptibility and/or responsiveness to AMF were found among wheat genotypes differing in ploidy number, geographic origin, nutrient use efficiency and year of release^16,18,21,22^. From an ecological point of view, wheat breeding should be pursued by selecting varieties showing high mycorrhizal susceptibility, as arbuscular mycorrhizal (AM) fungal root and soil colonization provide many non-nutritional benefits and agroecosystem services, e.g. plant tolerance to biotic and abiotic stresses, the maintenance of soil inoculum potential and soil structure, even in the absence of growth responses^2^.

Little information is currently available on the genetic diversity for AMF susceptibility among genotypes of wheat in general and, particularly, of durum wheat (*Triticum turgidum* ssp. *durum*)^19,23^, a worldwide grown crop at the base of the pasta industrial chain. Some studies have reported about the susceptibility to AMF of selected accessions of cultivated and diploid ancestors, nevertheless only recently an association study on a significant number of genotypes of bread wheat (*Triticum aestivum*) was conducted^24^. A significant variation in susceptibility to AMF was reported among a few durum wheat varieties^23^, stressing the importance of screening a high number of genotypes, to assess the genetic variability of wheat ability to be colonized by AMF.

Here, we report on a study designed (*i*) to evaluate the mycorrhizal status of 108 lines of *T*. *turgidum* ssp. *durum*, differing for origin and year of release, using two AM fungal species (*Funneliformis mosseae* and *Rhizoglomus irregulare)* and (*ii*) to detect genetic markers in linkage with chromosome regions involved in AM fungal root colonization through genome wide association analyses.

## Materials and Methods

### Plant and fungal materials

Plant material (Table S1), supplied by the CREA Research Centre for Cereal and Industrial Crops (Foggia, Italy), was part of a tetraploid wheat (*Triticum turgidum*, 2n = 4x = 28; AABB genome) collection extensively detailed by Laidò *et al*.^25,26^.

The genotypes used (number of accessions in brackets) were: wild emmer [*Triticum turgidum* ssp. *dicoccoides* (9)], emmer [*Triticum turgidum* ssp. *dicoccum* (13)] and, durum wheat [*Triticum turgidum* ssp. *durum* (108)]. The 108 accessions of durum wheat, previously genotyped by SSR and DArT markers as described in Laidò *et al*.^25^, were characterized in this study with the 90 K Infinium iSelect array containing 81.587 highly-informative gene-associated SNP markers^27^.

Two different experiments were set up. In the first one (Exp. 1) the fungus *Funneliformis mosseae* (T.H. Nicolson & Gerd.) C. Walker & A. Schüßler, isolate IMA1, was used to explore variability for AM colonization in the three subspecies. In the second experiment (Exp. 2) two isolates were used in two independent trials for AMF colonization evaluation: *F*. *mosseae* isolate IMA1 and *Rhizoglomus irregulare* (Błaszk., Wubet, Renker & Buscot) Sieverd., G.A. Silva & Oehl, isolate IMA6. Isolates were obtained from pot-cultures maintained in the collection of the Microbiology Laboratories of the Department of Agriculture, Food and Environment, University of Pisa, Italy.

The AM fungal inoculum used in each experiment was produced in greenhouse, by growing *Trifolium alexandrinum* L. in 8.0 L plastic pots containing a mixture (1:1, by volume) of soil and calcined attapulgite clay (OILDRI, Chicago, IL). The soil was a sandy loam collected at the University farm, near S. Piero a Grado (Pisa). Chemical and physical characteristics of the soil used were as follows: pH_(H2O)_, 8.0; clay, 15.3%; silt, 30.1%; sand 54.5%; organic matter, 2.2% (Walkley-Black); extractable P, 17.6 mg kg^−1^ (Olsen); extractable K, 149.6 mg kg^−1^. The mixture was steam-sterilized (121 °C for 25 min, on two consecutive days), to kill naturally occurring AMF. Each pot was inoculated with 2 L (25% of total pot volume) of a crude inoculum (mycorrhizal roots and soil containing spores and extraradical mycelium) of each isolate. After five months, the soil and the root systems were removed from the pots and air dried. Then, the roots were cut, carefully mixed with the soil and stored at room temperature, until use.

### Experimental setup

Seeds of the accessions of *Triticum* species were sown in 6 cell HDPE propagation flats (2–3 seeds per each 50 ml cell), which were arranged, every eight flats, in trays. Cells were filled with a mixture of the AM fungal crude inoculum, prepared as described above, and sterilized calcined attapulgite clay, 1:1 by volume. Plants were thinned to one per cell at emergence, grown in a unheated greenhouse and watered with tap water when needed. Nutrients were supplied by adding 5 mL of a low P nutrient solution once a week to each cell, up to the end of the experiment. The nutrient solution contained NH_4_NO_3_ (40 mg/L), KNO_3_ (102 mg/L), KH_2_PO_4_ (0.5 mg/L), K_2_SO_4_ (14 mg/L), KCl (11 mg/L) from commercial grade compounds. Trays were randomly moved every week, to avoid position effects. Seventy days after emergence, plants were removed from cells and roots were separated and analysed for mycorrhizal colonization. The same scheme was followed for both Exp. 1 and Exp. 2. Exp. 1, carried out from November 2009 to January 2010, was aimed at evaluating the extent of early AMF colonization produced by *F*. *mosseae* in 94 tetraploid wheat accessions (of which 72 of *T*. *durum*, 13 of *T*. *dicoccum*, and 9 of *T*. *dicoccoides*).

Due to the highly variable mycorrhizal susceptibility observed among durum wheat genotypes during the first experiment, Exp. 2, carried out from November 2011 to January 2012, was aimed at expanding the evaluation of AMF colonization to a larger set of *T*. *durum* accessions (for a total of 108) and to an additional AM fungus, *R*. *irregulare*.

Throughout the growing period, greenhouse temperatures were similar in the two experiments, with the exception of the first twenty days of December 2009, which were much colder than in 2011 (outdoor temperature: average minimum and maximum 0 °C and 10 °C in 2009, and 6 °C and 15 °C in 2011).

### Mycorrhizal assessment

Mycorrhizal colonization was assessed by clearing roots with 10% KOH in a 80 °C water bath for 15 min and staining with Trypan blue in lactic acid (0.05%) after 10 min in 2% aqueous HCl. Percentages of AM colonization were estimated under a dissecting microscope (Wild, Leica, Milano, Italy) at 25x or 40x magnification by the gridline intersect method^28^. In order to assess the colonization pattern, samples of colonized roots were mounted on slides and observed at magnification of 125x and 500x under a Polyvar light microscope (Reichert-Jung, Vienna, Austria).

### Phenotypic data analysis

Data are presented as means and standard errors of means of three replicates. A nested analysis of variance (ANOVA), with accessions nested within *Triticum* species as random factors, was carried out on data of Exp. 1. Variance partitioning was performed to show which hierarchical level (species or accession) contributed the most to AM fungal colonization variation^29^.

ANOVA was also carried out on data harvested from the durum wheat accessions inoculated in both independent trials of the Exp. 2, to test the genotype effect on AM fungal root colonization. All data were arcsin transformed to fulfil the assumptions of the ANOVA. Pearson correlation coefficients were calculated to determine the relationship between root colonization by *F*. *mosseae* and *R*. *irregulare* and between root colonization and year of release.

### Durum wheat genetic structure analysis

The molecular characterization of the tetraploid wheat genotypes is fully described by Laidò *et al*.^25^. In the present study the population structure was determined on the 108 durum wheat varieties. The molecular data, cleaned from failed data (missing data) and alleles that occurred at a low frequency (p < 0.9) (26 SSR, 649 DArT and 12,225 SNP markers), were processed using the STRUCTURE program, version 2.2 (http://pritch.bsd.uchicago.edu/ structure.html)^30^. The number of sub-groups (K) was estimated by 20 independent runs for each K (from 2 to 20) applying the admixture model, with allele frequencies uncorrelated for SSRs and correlated for DArT and SNP markers, 100,000 Markov Chain Monte Carlo (MCMC) repetitions, and a 100,000 burn-in period. The means of the log-likelihood estimates for each K were calculated. The true K was determined using both an estimate of the posterior probability of the data for a given K (as proposed by Pritchard *et al*.^30^), and the Evanno ∆K^31^. A genotype was considered to belong to a group if its membership coefficient was ≥0.50^32^.

### Detection of QTLs by GWAS

The association mapping analysis was carried out on 108 durum wheat genotypes on both AM fungal species used in the Exp. 2. The marker-phenotype association analysis was based on the polymorphisms present in 12,225 SNPs with a minimum allele frequency >0.10. We used the mixed linear model (MLM) methods within TASSEL, based on the kinship matrix (MLM + K) and the MLM based on both the K-matrix and the Q-matrix (MLM + Q + K), to test for possible Marker Traits Associations (MTAs) both taking into account only population structure and/or kinship information. The trimmed marker SNP dataset was used to generate a marker similarity matrix containing all the lines (*K* matrix) using the TASSEL software. TASSEL calculates the kinship as the proportion of alleles shared between each pair of lines. Once this matrix was calculated, the numbers were rescaled, so that they were between 0 and 2^33^. We also used a compressed mixed linear model (CMLM) as proposed by Zhang *et al*.^34^ and SUPER model proposed by Wang *et al*.^27^ implemented in GAPIT, the last one increasing statistical power, run time and memory use. Finally, results from all different methods were compared, and those showing the best Quantile-quantile (Q-Q) plots were chosen for further discussion. The critical *P* values for the assessment of the significance of the MTAs were calculated separately for each AM fungal species in the Exp. 2, based on false discovery rate (FDR) of 0.05, which is defined as the expected proportions of the true null hypotheses that are rejected. The algorithm described by Benjamini & Hockberg^35^ was used because it was shown to control FDR for independent test statistics, but also for some types of positive dependence. FDR correction, applied on P-values obtained from MLM, CMLM and SUPER analysis, did not produce significant results. Therefore a significance level for the SUPER output was set as α = 0.05 (without adjustment). However, the deviation of the observed −log10(P) values from the expected distribution (see Q-Q plots in Fig. S2) and the high number of significant MTAs associated with mycorrhizal colonization of both AM fungal species could indicate the detection of numerous false-positives. To provide more confidence in the associations for AM fungal root colonization detected using this model and to minimize possible false-positives we reported as associated markers only the common MTAs identified in each individual AM fungal species of the Exp.2 and grouped into a single putative QTL. All of the significant MTAs, identified using the SUPER model, located on the durum wheat consensus maps^36,37^ within short map intervals (5–10 cM) were grouped into a single putative QTL.

### Identification of candidate genes

The sequences of the markers present in the Confidence Interval (CI) of the QTLs identified in this study, were subjected to a BLASTN search on the wild emmer wheat Zavitan genome (Zavitan WEWSeq v.1.0), implemented in the Graingenes website (https://wheat.pw.usda.gov/GG3/wildemmer_blast), for gene annotation within the corresponding interval in the physical map^38^. The threshold E-10 has been considered to identify candidate genes for root colonization.

## Results

### Phenotypic variability

Light microscope observations of colonized roots showed the presence of *Arum*-type colonization pattern, characterized by rapid spread of the fungus via the apoplastic space between cortical cells of the root parenchyma and by the production of many intercellular hyphae and dense patches of arbuscules in contiguous cortical root cells. Intercellular and intracellular vesicles were also detected.

**Figure 1:**
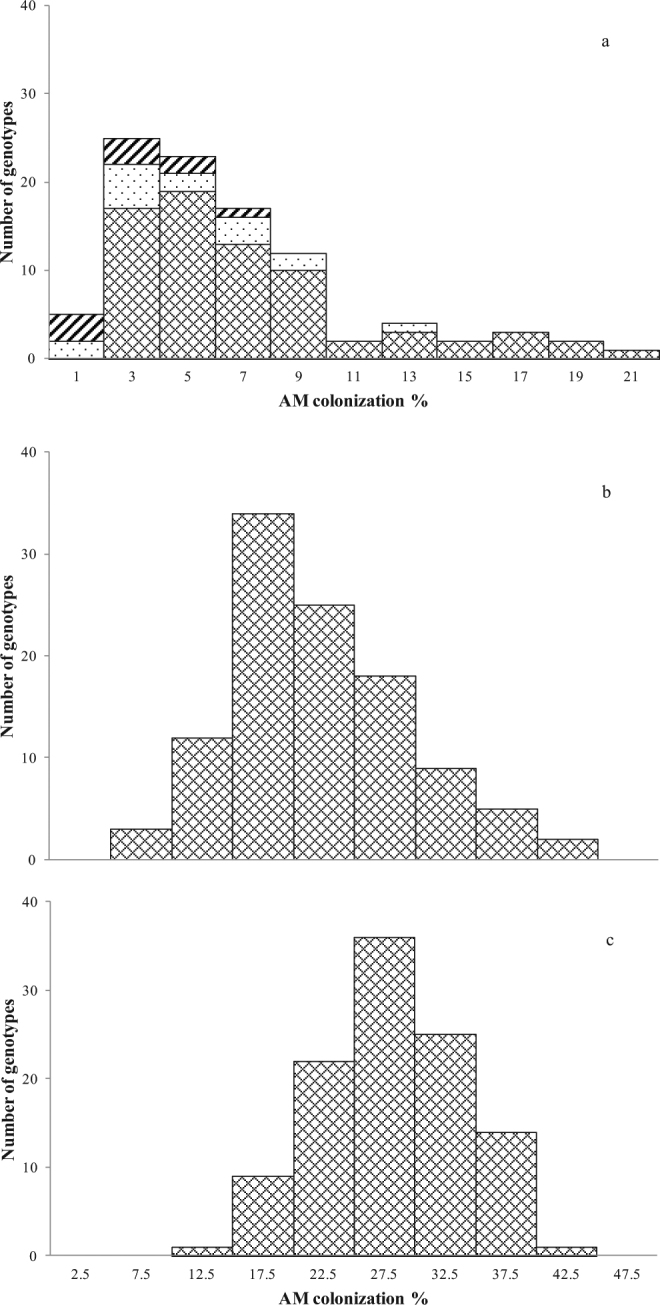
Frequency distribution of arbuscular mycorrhizal (AM) colonization in tetraploid wheat genotypes at 70 days after emergence. (**a**) Colonization of *Triticum turgidum* ssp. *durum* (crosshatched bars) *T*. *turgidum* ssp. *dicoccum* (dotted bars) and *T*. *turgidum* ssp. *dicoccoides* (hatched bars) by *Funneliformis mossseae* in Exp. 1. (**b**) Colonization of *T*. *turgidum* ssp. *durum* (cross hatched bars) by *F*. *mosseae* in Exp. 2. (**c**) Colonization of *T*. *turgidum* ssp. *durum* (cross hatched bars) by *Rhizoglomus irregulare* in Exp. 2.

In Exp. 1, accessions (72) of durum wheat and accessions belonging to wild (9) and domesticated (13) emmer were compared. A high variability in root colonization was observed in all wheat sub-species tested (range: 2.2–21.2% in durum wheat; 1.0–12.0% in domesticated emmer; 0.7–7.1% in wild emmer) (Fig. 1a), with an upward shift in the mean level of root colonization from wild (3.5%) and domesticated emmer (5.2%) to durum wheat (7.4%). Nested analysis showed that most of the variation was allocated at the accession level (45%), supporting the presence of substantial differences among the accessions for this trait, while sub-species contributed only 15% to total variation (Table 1). The genotypes that showed the lowest root colonization values mainly belonged to wild and domesticated emmer, i.e. wild emmer MG4328/61, PI355459, MG29896 and cultivated emmer Farro Molisano, Farro Garfagnana, and Farro Italia Centrale (Supporting Information Fig. S1a).

**Table 1:** Percentage of colonized root length, mean and standard error (SE), of tetraploid wheat accessions by the arbuscular mycorrhizal fungus *Funneliformis mosseae* and nested ANOVA results of the effect of species and accession in Experiment 1. Mean in the same column followed by different letters differ significantly (Tukey’s test, p < 0.05).

		Mean		SE		
*Triticum turgidum* ssp. *durum*		7.4 a		0.5		
*Triticum turgidum* ssp. *dicoccum*		5.2 b		0.9		
*Triricum turgidum* ssp. *dicoccoides*		3.5 b		0.7		
**Source**		**Df**	***F***	***P*** **-value**	**Variance component**	**% Variance component**
Species	Hypothesis	2	6.6	0.002	6.288	16.0
	Error	92.8				
Accession	Hypothesis	93	8.9	<0.001	19.139	48.6
	Error	458			13.976	35.5

Root colonization ranged between 2 and 10% in 82 durum genotypes tested in Exp. 1, including all indigenous landrace populations from Sicily, which ranged from 2.7 and 8.1%. Among modern varieties, Primadur, Parsifal, Preco, San Carlo, and PR22D89 showed the lowest mycorrhizal colonization values (2.2–2.5%), while values greater than 15% were found in Grecale, Gianni, Maestrale, Ciccio and Claudio, genotypes derived from Italian breeding programs and in Durfort (Fig. S1a).

In Exp. 2 we assessed the level of root colonization of 108 selected durum wheat genotypes, inoculated with either *F*. *mosseae* or *R*. *irregulare* (Figs 1b,c and S1b,c). The average level of mycorrhizal colonization by *F*. *mosseae* was 22.1%, more than 3 times higher than in Exp. 1 (6.6%). About 15% of all genotypes tested showed a colonization values lower than 15%, while a large majority (70%) showed values between 15 and 30%. Iride and Saragolla showed the highest values (43**–**44%), while Vendetta, Baio, L252 and Ceedur the lowest (below 10%). The average level of root colonization by *R*. *irregulare* was 28.0%. In most genotypes (about 65%) mycorrhizal colonization was between 15 and 30%. The Italian released varieties Gianni, Maestrale, Zenit, Valnova, Valforte, Platani, Ofanto, Normanno, Antas and Ancomarzio were highly colonized by *R*. *irregulare* (>35%), together with Saadi (a French breed), Pedroso (a Spanish breed), and UC1113 (a Californian breed). The lowest mycorrhizal colonization was found in Ceedur, a French breed which also performed very poorly with *F*. *mosseae* (Fig. S1b,c).

The comparison of durum wheat susceptibility to *F*. *mosseae vs*. *R*. *irregulare* showed that colonization levels in *R*. *irregulare* were higher than in *F*. *mosseae* in 86 out of 108 accessions, though statistical analyses revealed a low correlation (*r* = 0.21; *P* = 0.028). The variety UC1113 showed the highest level of root colonization with both fungi (39 and 38%, respectively), followed by Saadi, Fauno, Simeto, Varano and Durfort (>30%). Other accessions showed very similar levels of colonization with the two fungal isolates, e.g., Appio (20.6%), Valgerardo (26.6% *vs*. 27.2%), Simeto (33.7% *vs*. 34.4%) and Grifoni 235 (28.8% *vs*. 27.9%). On the contrary, a small group of accessions, despite the high susceptibility to *F*. *mosseae* (>30%), showed lower levels of root colonization in the presence of *R*. *irregulare*, e.g. Adamello (36.0% *vs*. 16.7%), 5-BIL 42 (33.0% *vs*. 18.5%) and Claudio (33.5% *vs*. 18.9%). On the contrary, a large group of accessions showed a higher susceptibility to *R*. *irregulare* than to *F*. *mosseae*, e.g. L252 (29.4% *vs*. 9.6%), Baio (28.3% *vs*. 9.4%) and Vendetta (28.5%*vs*. 10.0%).

**Figure 2:**
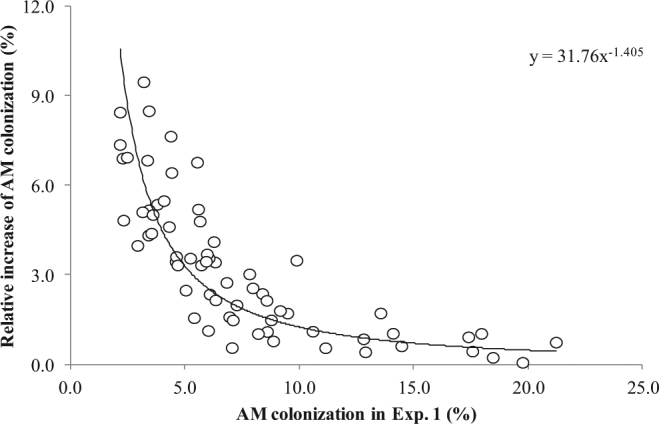
Relationship between arbuscular mycorrhizal (AM) colonization of *Funneliformis mosseae* in *Triticum turgidum* ssp. *durum* 70 days after emergence in Exp. 1 and the relative increase of AM colonization observed in Exp. 2.

Interestingly, analyzing the susceptibility of durum wheat genotypes to *F*. *mosseae* in the two experiments, the increases in colonization levels detected in Exp. 2, compared with the values found in Exp. 1 were approximated by a power law functional relationship (*r* = 0.84; *P* = 0.01) (Fig. 2). Indeed, some accessions showed higher mycorrhizal colonization values (more than 7 times) in Exp. 2, such as Simeto (from 3.2 to 33.7%), Trinakria (from 3.5 to 32.8%), Parsifal (from 2.2 to 20.7%) and Primadur (from 2.19 to 18.3%). On the other hand, the smallest increases (less than 50%) were observed in Ciccio, Dylan, Maestrale and Gianni, whose root colonization in Exp. 1 ranged from 12.9% to 19.8%. In both cases these varieties did not share either a common pedigree or a similar genetic structure. However, some accessions showed increases larger than expected by the mentioned relationship, *e*.*g*. UC 1113 (from 4.4 to 38.1%), Saragolla (from 5.6 to 43.3%), and Iride (from 9.9 to 44.5%), while Tito, Cappelli and PR22D89 showed increases lower than expected. In addition, a low correlation (*r* = 0.25; *P* = 0.043) was found in *F*. *mosseae* root colonization between Exp. 1 and 2.

A low but significant correlation was observed between levels of colonization and year of release of the different accessions in Exp. 1 (*r* = 0.28; *P* = 0.019; n = 72). Though, considering only modern varieties (that is, those released after 1970), no effect of the year of release on susceptibility to *F*. *mosseae* was detected (*r* = 0.20; *P* = 0.131; n = 64), as mycorrhizal colonization percentages ranged from 2.2 to 21.2. Likewise, the relationship between levels of colonization and year of release proved non significant for accessions tested in Exp. 2, both for *F*. *mosseae* (*r* = 0.009; *P* = 0.930) and *R*. *irregulare* (*r* = 0.096; *P* = 0.331).

### Genetic structure of durum wheat accessions

**Figure 3:**
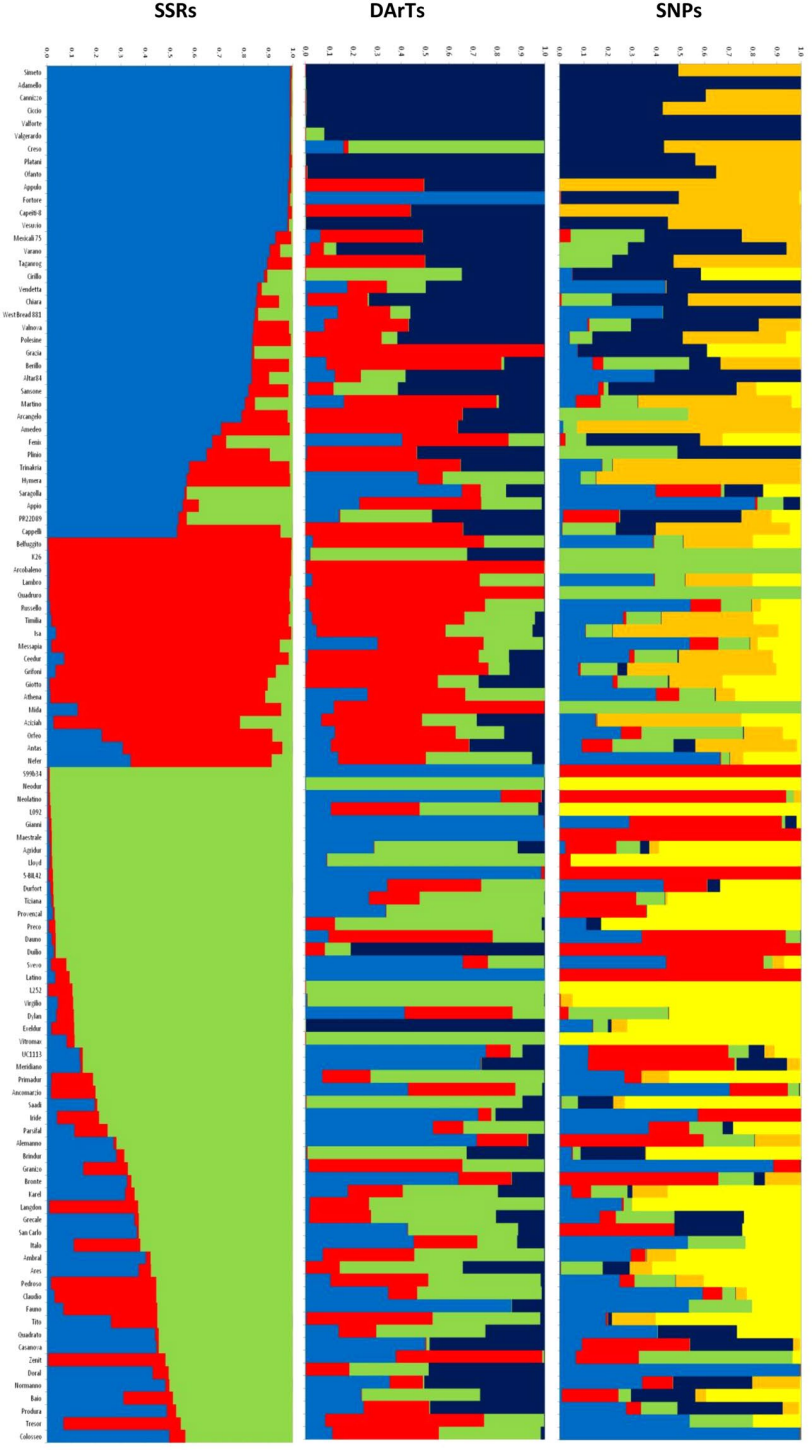
Population structure of the 108 durum wheat accessions using SNP markers.

Before proceeding with association mapping, a genetic structure analysis was conducted using a Bayesian approach on 108 durum wheat varieties implemented in the STRUCTURE software. Following the method of Evanno *et al*.^31^, the maximum ΔK occurred at K = 3 for the SSRs, K = 4 for the DArTs and K = 6 for the SNP markers. The structure at K = 3 identified with SSR non correlated markers was used for association mapping analysis. At K = 3 with the SSR markers the collection was split in three sub-groups containing 37, 18 and 49 accessions, plus four varieties admixed that were included in all groups (Fig. 3). Durum wheat accessions were clustered into three large groups (group 1, group 2 and group 3). In particular, durum wheat varieties grown or released at the beginning of the last century (Cappelli, Grifoni 235, Taganrog) and those selected in Italy until the end of the ‘80 (Val- series, Arcangelo, Duilio, Simeto) fell into group 1. Group 2 consisted of old varieties (*i*.*e*. Sicilian origin as Russello and Timilia) and those derived from introgression with wild and domesticated wheat (Lambro and Belfuggito) or other exotic material, while the most recent varieties derived from the breeding work in various countries were clustered in group 3. Therefore, results of genetic structure analysis of durum wheat varieties were in agreement with their origin and year of release, confirming our previous study^25^.

### Association mapping

The marker-phenotype association analyses were based on polymorphisms of 12,225 SNP markers. Regarding significant associations, all data for each MTA identified using SUPER model are shown in Table S2 and mapped according to the durum wheat consensus maps reported by Marone *et al*.^36^ and Maccaferri *et al*.^37^.

Seven QTLs in common to both AM fungi, *F*. *mosseae* and *R*. *irregulare*, obtained using AMF colonization values of Exp. 2, were identified. Overall, these loci detected 7 putative QTLs linked with durum wheat mycorrhizal susceptibility and located on chromosomal groups 1A, 2B, 5A, 6A, 7A and 7B with *r*^2^ values ranging from 7% to 16% (Table 2). The QTL regions, identified by multiple significant associations, were defined by sets of closely linked SNP markers significantly associated with phenotype, located at very few cM. For each of the QTLs that were identified as linkage blocks of adjacent markers, all the markers significantly associated with the phenotype were checked for consistency of their effects and the marker with the most significant association was considered as the QTL-tagging marker.

**Table 2:** Significant marker trait associations (MTAs) and corresponding quantitative trait loci (QTLs) for root colonization of durum wheat (*Triticum turgidum* subsp. *durum*) by arbuscular mycorrhizal fungi.

QTLs	Marker	Chr.	Pos. (cM)	*P*-value MLM Model (Q + K)	*R*^2^ (%)	Other MTAs in the region for single fungus	Range *R*^2^ (%)	Chr. Interval (cM)
QTamf-1A	SNP_72794	1A	19,7	*Fun*. 1,3E-02	8	5	7–9	1,1
				*Rhiz*. 2,1E-03	12	45	8–15	10,2
QTamf-2B	SNP_34450	2B	181,6	*Fun*. 2,3E-02	7	1	7	0,0
				*Rhiz*. 3,4E-04	16	12	7–11	5,6
QTamf-5A	SNP_29990	5A	0,1	*Fun*. 1,6E-02	8	—	—	—
				*Rhiz*. 2,4E-02	8	11	7–10	0,7
QTamf-6A.1	SNP_76143	6A	21,4	*Fun*. 3,4E-03	11	1	10	0,0
				*Rhiz*. 5,1E-03	10	—	—	—
QTamf-6A.2	SNP_76302	6A	97,4	*Fun*. 2,1E-02	7	—	—	—
				*Rhiz*. 2,6E-02	8	—	—	—
QTamf-7A	SNP_75676	7A	139,4	*Fun*. 2,0E-02	7	1	12	3,8
				*Rhiz*. 1,7E-02	8	1	8	0,0
QTamf-7B	SNP_35797	7B	112,5	*Fun*. 2,0E-02	7	2	8	0,0
				*Rhiz*. 1,7E-02	8	2	7–9	0,0

Common MTAs identified in both fungal species are reported.

Chr., chromosome; Pos., position on the chromosome in cM; *R*^2^, variance explained by marker in percentage (%).

### Candidate genes for arbuscular mycorrhizal colonization

Sequences of SNP markers related to MTAs identified in this work on chromosomes 1A, 2B, 5A, 6A, 6B, 7A and 7B and those of markers included in their CI within 3 cM, were projected to the wild emmer wheat Zavitan genome (https://wheat.pw.usda.gov/GG3/wildemmer_blast). Based on similarity search results, putative functions are proposed for three MTAs (Table 3; Table S3). Other two putatively expressed sequences corresponded to protein sequences without a functional annotation or known domains, whereas no hits were found for the remaining two MTAs. Moreover, putative functions were assigned to the most markers included in the CIs of QTLs. Different functional categories have been found. Putative proteins involved in stress conditions, plant growth and development processes, cellular homeostasis, biosynthesis of secondary metabolites, pathogen resistance but also splicing and translation mechanisms have been identified.

**Table 3:** Candidate genes corresponding to the MTAs identified in this study as associated with the ability of wheat roots to establish arbuscular mycorrhizal symbiosis, and QTLs related to the trait from the literature, mapping in the same chromosome regions.

NameQTL	Chr.	Peak marker	BLAST results	Marker name - MTA/QTL related (Reference)
QTamf-1A	1A	SNP_72794	F-box/RNI-like superfamily protein	wPt-4666 QPm66–1A (Ben David *et al*.^57^); IWB4497 TRN (Maccaferri *et al*.^58^)
QTamf-2B	2B	SNP_34450	K(+) efflux antiporter 2, chloroplastic	wPt-7360 Sr (Laidò *et al*.^26^), gwm4828 QPm66 (Ben David *et al*.^57^), Barc159 MlZec (Marone *et al*.^59^), wmc441-wmc149 Yr53 (Xu *et al*.^60^); IWB54597 PRL,TRL (Maccaferri *et al*.^58^); IWB1384 RDW, RGA (Maccaferri *et al*.^58^)
QTamf-5A	5A	SNP_29990	Unknown function	IWB7361 TRL (Maccaferri *et al*.^58^); IWA1062 Lr (Aoun *et al*.^61^)
QTamf-6A.1	6A	SNP_76143	60 S ribosomal protein L13-1	wPt-7623 QPm (CP1) (Marone *et al*.^59^); IWB64435 RDW, RT6 (Maccaferri *et al*.^58^); IWA1749 Lr (Aoun *et al*.^61^)
QTamf-6A.2	6A	SNP_76302	no hits found	IWA3024 Lr (Aoun *et al*.^61^); tPt-4209 Sr (Laidò *et al*.^26^); tPt-4209 Sr (Letta *et al*.^62^); IWB50538 TRL (Maccaferri *et al*.^58^)
QTamf-7A	7A	SNP_75676	Unknown function	IWA477 Lr (Aoun *et al*.^61^); Barc121 Sr22 (Haile *et al*. 2012); IWB43304 QFRi.mgb (Giancaspro *et al*. 2016); IWB35428 RGA (Maccaferri *et al*.^58^); Barc121 FHB (Kumar *et al*. 2007); wPt-8399 FHB (Ghavami *et al*.^64^); IWA2752 RGA, IWB71703 TRL (Maccaferri *et al*.^58^)
QTamf-7B	7B	SNP_35797	no hits found	wPt-4025 FHB (Ghavani *et al*. 2011); gwm274 QLr.gpg (Desiderio *et al*. 2014); IWB11767 Qyrdurum (Liu *et al*.^70^)

NA, sequence not available; Pm, Powdery mildew; TRN, Total Root Number; Sr, Stem rust; MlZec, Powdery mildew; Yr, Yellow rust; PRL, Principal Root Length; TRL, Total Root Length; RDW, Root Dry Weigth; RGA, Root angle; LRN, Lateral Root Number; Lr, Leaf Rust; RT6, Trait Root-Related; ARL, Axial Root Length; FR, Fusarium Resistance; FHB, Fusarium Head Bligth.

## Discussion

This work represents the first study carried out using a large collection of wild and domesticated tetraploid wheat accessions of different backgrounds and origins, showing large variations of AM fungal colonization by two fungal symbionts and the occurrence of genetic markers associated to such a trait in durum wheat.

### Phenotypic variability

We found large differences, in terms of percentage of AM colonization, across the tetraploid wheat accessions tested, in contrast to previous works reporting low variability in the level of AM colonization in wild^22^ and cultivated tetraploid wheat^17,19^. However, differences between the tetraploid wheat species *Triticum timopheevii* var. *araraticum* belonging to the AAGG group and the tetraploid wheat with the AABB genome, were reported^19^, suggesting some genetic variability for mycorrhizal colonization levels. Our data, obtained analysing 108 durum wheat accessions, support and extend previous findings obtained on five durum wheat varieties, selected among five genetically characterized groups^23^.

In general, the level of mycorrhizal colonization is affected by different environmental variables, such as light intensity, whose decrease inhibits mycorrhizal development^39,40^, and temperature, whose increase (up to 30 °C) enhances root colonization^41,42^. Considering the temperature conditions of the experiments, the higher levels of root colonization by *F*. *mosseae* in Exp. 2 may be ascribed to the higher temperature values recorded in the second experimental year. In addition, the different responses of AMF to the higher temperature conditions were not consistent among the durum wheat genotypes tested, suggesting a complex genetic control of root colonization. Other factors intrinsic to plant genotypes and variable in the diverse accessions, such as root morphology and exudation, root to shoot ratio or mineral nutrient concentration are known to affect rhizosphere microrganisms^43,44^ and the degree of mycorrhizal colonization^6,45–47^.

The different durum wheat accessions did not show consistent levels of root colonization by *F*. *mosseae* and *R*. *irregulare*, as confirmed by the relatively low values of correlation coefficient detected (*r* = 0.21), in agreement with previous data^16^, underlining that the identity of fungal symbionts is a key factor affecting the development of intraradical fungal growth. Although single isolates investigations may have limited relevance to field plant-AMF community interactions, they provided quantitative data on isolate traits affecting AMF root colonization, not achievable with AMF natural communities, which are, so far, quantitatively untraceable. Further works may reveal the role played by synthetic assemblages in the establishment of mycorrhizal colonization, as the result of competitive, synergistic or antagonistic interactions among the relevant AMF taxa.

The durum wheat genotypes included in this study encompass accessions released during a large time span, making it possible to analyse the effects produced by genetic improvement. Our data show that a century of breeding did not select against mycorrhizal colonization, in agreement with other authors reporting that modern plant breeding programs did not lead to the suppression of AM fungal colonization in wheat^20^ and in maize^48^.

Considering that durum wheat genotypes analysed in the two experimental years showed a clear genetic structure, essentially linked to the origin and year of release^25^, our results suggest the absence of relationships between colonization levels and breeding activity. Moreover, we can speculate that the introduction of gene *Rht* from Japanese bread wheat variety Norin-10 to durum wheat, associated with a semi-dwarf growth habit in modern wheat varieties^49^, has not affected their response to AM symbiosis. Therefore new breeding programs could be implemented for this trait, as no direct or indirect selective pressure seems to have been exerted.

### QTL analysis for arbuscular mycorrhizal colonization

In Exp. 2, considering each AM fungal species independently, a large number of MTAs were identified (Table S2), indicating the complexity of the genetic control of this trait at an early growth stage. However, to provide more confidence in the analysis of associations for AM fungal root colonization, only the common MTAs identified between the two AM fungal species and grouped into a single putative QTL were considered. Our results show a good genetic basis for mycorrhizal colonization trait confirming previous studies, in which a heritable genetic basis^17^ with a limited number of QTLs was identified^50^. An early work on bread wheat^51^, carried out using a set of intervarietal chromosome substitution lines, indicated six different chromosomes as possibly responsible for positive effects on mycorrhizal response trait, with homeologous groups 5 and 7 in the B and D genomes having the largest effects.

Very recently, chromosomes 3A, 4A, 5A and 7A have been found to be associated to root colonization by mycorrhizal fungi in wheat^24^. The chromosomes 5A and 7A were in common with that reported in our study, nevertheless the region identified on chromosome 5A in this study was different, as the SNP_29990 (QTamf-5A) was at more than 50 cM far from the marker Ex_c5445_981 mapped by those authors. Moreover, the MTA here identified and located on chromosome 7A (SNP_75676, QTamf-7A) was at 20 cM from that mapped in bread wheat (BS00064143_51), suggesting that the region could be the same.

The comparison of our results with those reported in the literature, based on common markers, indicates that some MTAs here identified co-localized or were very close to QTL for root morphological traits, suggesting that some feature of root morphology and structure can influence plant-AMF interactions (Table 3). Actually many studies showed that root architecture, in particular lateral and adventitious root formation, is affected by AMF colonization^52–54^. On the other hand, MTAs co-localized or were very close to QTL/genes for disease resistance (Table 3). In studies exploring plant transcriptomes, some genes regulating pathogenic host interactions were reported to be involved in the establishment of AMF root colonization^55,56^. So, it was not surprising in this work to find many chromosome regions that co-mapped with QTL linked to wheat disease resistance genes. The QTamf-1A was very close to the SSR CFA2158, marker peak of a QTL for powdery mildew resistance^57^ and at 10 cM from a QTL explaining total root number (TRN) by Maccaferri *et al*.^58^.

Different QTL/resistance genes have been mapped in the same region of the MTA SNP_34450 located on chromosome 2B, in particular the marker wPt-7360 associated to stem rust resistance^26^; the QTL QPm66 with the peak marker GWM4828, and the gene MlZec tagged by the SSR BARC159 for powdery mildew resistance^57,59^; the gene Yr53 for yellow rust resistance mapped in the marker interval WMC441-WMC149 by Xu *et al*.^60^. Moreover, QTLs for root traits, such as length of principal root (PRL), total root length (TRL), root dry weight (RDW) and root angle (RGA), have been found in durum wheat in the same region^58^. A correspondence between the SNP_29990 on 5A and two QTLs for TRL^58^ and leaf rust resistance^61^, respectively, was also found.

The two MTAs involved in mycorrhizal colonization mapped on the chromosome 6A corresponded, the first one (21.4 cM) to QTLs for root traits (RDW, RT6^58^) and disease resistance (powdery mildew and leaf rust^59,61^), whereas the second one (97.4 cM) corresponded to QTLs for TRL (IWB50538^58^) and for resistance against stem rust and leaf rust (tPt-4209^62^, 2013 and^26^; IWA3024^61^). Moreover, in the region of the second MTA on 6A Marone *et al*.^59^ reported two DArT markers (wPt-8331, wPt-3191) for which a putative function of NBS-LRR genes has been indicated. A very recent study on QTL mapping for root traits in a durum wheat x *T*. *dicoccum* population and meta-analysis reported a meta-QTL including QTL for RDW, TRL and LRN that, based on common markers, could correspond to the region of the MTA SNP_76143 on chromosome 6A^63^.

Finally, a lot of MTAs associated to disease resistance have been mapped in durum wheat on chromosomes 7A and 7B where QTamf-7A and QTamf-7B for AM colonization were located. For example, the marker IWA477 linked to leaf rust resistance^61^ was at very few cM from the SNP_75676 on 7A, or the marker wPt-4025 associated to *Fusarium* head blight resistance identified in Tunisian-derived durum wheat populations^64^ was co-mapping with the SNP_35797 on 7B. Moreover, a meta-QTL that included two QTLs for root traits (MQTL21) has been reported by Petrarulo *et al*.^65^ in the region of chromosome 7B (wpt-0920) where the MTA for AM colonization was identified. A similar trend was found in other species. In poplar for example a QTL for rust resistance has been found to be located close to a QTL for ectomycorrhizal colonization^66^.

Functional analysis of the available sequences of mapped markers related to the MTAs and included in their CI, identified predicted proteins with a potential involvement in the physiological changes occurring after AM symbiosis establishment. For example, the MTA mapped on chromosome 1A corresponded to a putative F-box/RNI-like superfamily protein, activated in stress conditions in flowering plants and involved in protein-protein interaction, plant growth, cellular protein degradation, leaf senescence regulation^27^ and pathogen resistance^67^. Moreover, many resistance proteins or protein kinases or proteins having a role in oxidative stress-related pathologies have been also found in all the regions identified in this study. Previous studies also reported that markers associated to root colonization mainly code for genes involved in pathogen defence mechanisms or stress responses^11,24^. In recent years, information related to symbiotic genes or genes involved in different stages of the mycorrhizal colonization was available in literature^68^. Actually, we found two markers at 1 cM from the MTA on the chromosome 2B, corresponding to nodulin proteins, known to play a key role in the establishment of the symbiosis^69^. Moreover, different calcium-related genes have been identified in this study, well documented to be involved in mobilization and perception of the intracellular messenger by the AM fungus during symbiotic interactions with host roots^70^.

In our study, a large variability in AM colonization was found among the tested tetraploid wheat genotypes, confirming previous data obtained with other plants, such as legumes and trees crops^71,72^. In the tested genotypes, the ability to form AM symbiosis was not affected by genetic improvement, as most of the variability for this trait was found in free-threshing durum-type wheats, representing a source for the development of new genetic material of durum wheat.

The genetic mapping of mycorrhizal associated QTLs, here reported for the first time in durum wheat, will allow a more effective genetic manipulation of such a trait. The increasing availability of molecular resources for major crop species is driving new approaches to the understanding of crop development and the selection of future varieties, as sequence data in wheat will facilitate the generation of markers and the identification of candidate genes. Moreover, expression platforms are shifting trait measurements from purely morphological to gene expression levels, while QTL and association analyses are beginning to make the link between traits and the ruling relevant genes. Since the main aim, however, remains that of correlating phenotypic and genetic information, accurate assessments of relevant traits represent the basis of any successful study. Among plant phenotypic traits possibly related to AMF root colonization, root to shoot ratio^47^ and specific root length^73^ deserve further investigations. Recent technological progress will improve the integration of -omics approaches in order to understand phenotypic diversity and its underlying genetic variation, and to answer questions such as the role of plant and AM fungal genotypes in the development of AM symbiosis.

The results of this work emphasize the potential for further enhancement of root colonization through crossing and selection by using selected genotypes and exploiting the variability present in modern durum wheat genotypes without compromising the productivity of the new varieties.

## Electronic supplementary material

Supplementary information
