# Supplementary material for: Genetic markers associated to arbuscular mycorrhizal colonization in durum wheat
Source: Sci Rep. 2018 Jul 13;8:10612. doi: 10.1038/s41598-018-29020-6 (PMC6045686; doi:10.1038/s41598-018-29020-6)
Supplement: Supplementary file 1 — Supplementary information [file 41598_2018_29020_MOESM1_ESM.pdf]

Supplementary information

Genetic markers associated to arbuscular mycorrhizal colonization in durum wheat

Pasquale De Vita[1*], Luciano Avio[2*], Cristiana Sbrana[3*], Giovanni Laidò[1], Daniela Marone[1], Anna M. Mastrangelo[1,4], Luigi Cattivelli[5] and Manuela Giovannetti[2]

[1]Consiglio per la ricerca in agricoltura e l'analisi dell'economia agraria, Centro di Ricerca Cerealicoltura e Colture Industriali, S.S. 673 km 25+200, 71121 Foggia, Italy
[2]Dipartimento di Scienze Agrarie, Alimentari e Agro-Ambientali, Università di Pisa, Via del Borghetto 80, 56124 Pisa, Italy
[3]Istituto di Biologia e Biotecnologia Agraria CNR, Pisa, Italy
[4]Consiglio per la ricerca in agricoltura e l'analisi dell'economia agraria, Centro di Ricerca Cerealicoltura e Colture Industriali, Via Stezzano 24, 24126 Bergamo, Italy (present address)
[5]Consiglio per la ricerca in agricoltura e l'analisi dell'economia agraria, Centro di Ricerca Genomica e Bioinformatica, Via San Protaso 302, 29017 Fiorenzuola d'Arda (PC), Italy

Author for correspondence:
Pasquale De Vita
Tel: +0881-742972
Email: pasquale.devita@crea.gov.it

Supplementary Information

Figure S1. Arbuscular mycorrhizal (AM) colonization of *Triticum turgidum* ssp. *durum* 70 days after emergence in genotypes released before 1970, between 1970 and 1990, and after 1990

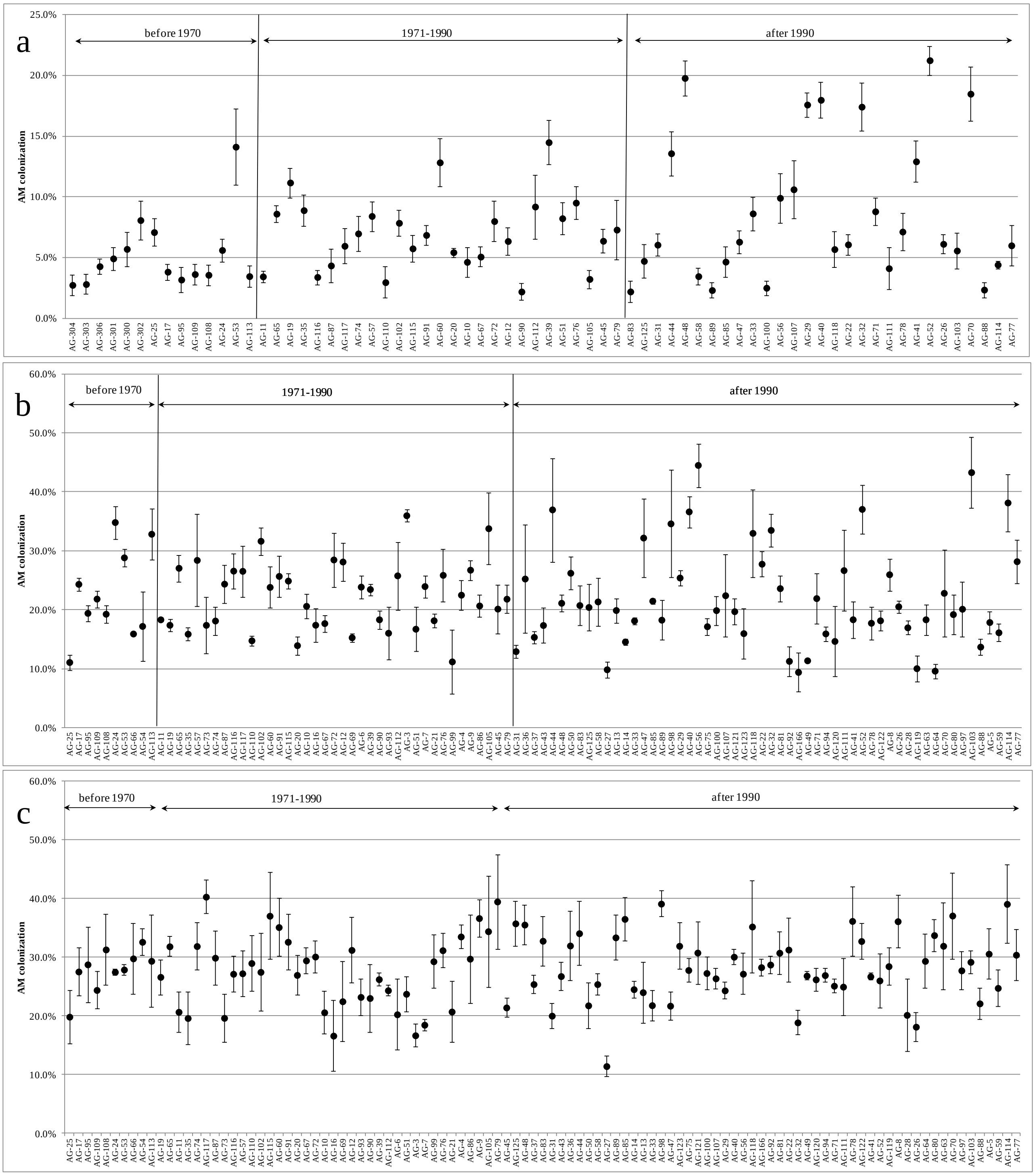
a
before 1970
1971-1990
after 1990
AM colonization
25.0%
20.0%
15.0%
10.0%
5.0%
0.0%
AG-304 AG-303 AG-306 AG-301 AG-300 AG-302 AG-25 AG-17 AG-95 AG-109 AG-108 AG-24 AG-53 AG-113 AG-11 AG-65 AG-19 AG-35 AG-116 AG-87 AG-117 AG-74 AG-57 AG-110 AG-102 AG-115 AG-91 AG-60 AG-20 AG-10 AG-67 AG-72 AG-12 AG-90 AG-112 AG-39 AG-51 AG-76 AG-105 AG-45 AG-79 AG-83 AG-125 AG-31 AG-44 AG-48 AG-58 AG-89 AG-85 AG-47 AG-33 AG-100 AG-56 AG-107 AG-29 AG-40 AG-118 AG-22 AG-32 AG-71 AG-111 AG-78 AG-41 AG-52 AG-26 AG-103 AG-70 AG-88 AG-114 AG-77
b
before 1970
1971-1990
after 1990
AM colonization
60.0%
50.0%
40.0%
30.0%
20.0%
10.0%
0.0%
c
before 1970
1971-1990
after 1990
AM colonization
60.0%
50.0%
40.0%
30.0%
20.0%
10.0%
0.0%

Supplementary Information

Figure S2. Cumulative distributions of observed P values for the SUPER model in the genome-wide association study in 108 wheat genotypes for Exp. 2 (Q-Q plots before and after FDR correction).

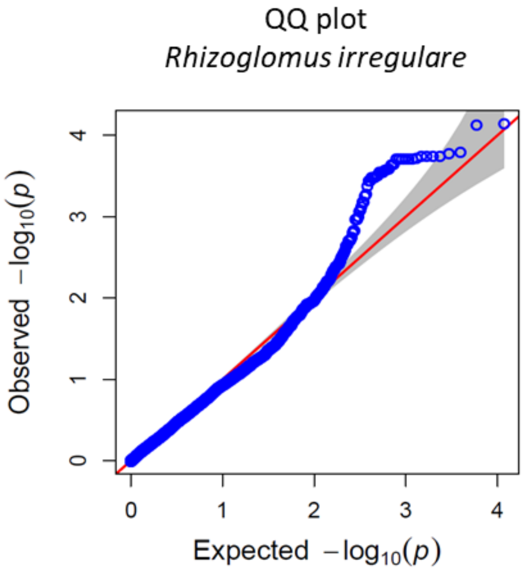
QQ plot
Rhizoglomus irregulare
Observed −log10(p)
Expected −log10(p)
0 1 2 3 4

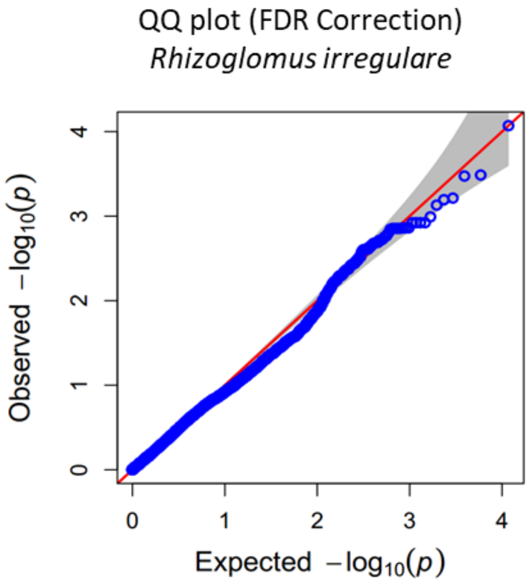
QQ plot (FDR Correction)
Rhizoglomus irregulare
Observed −log10(p)
Expected −log10(p)
0 1 2 3 4

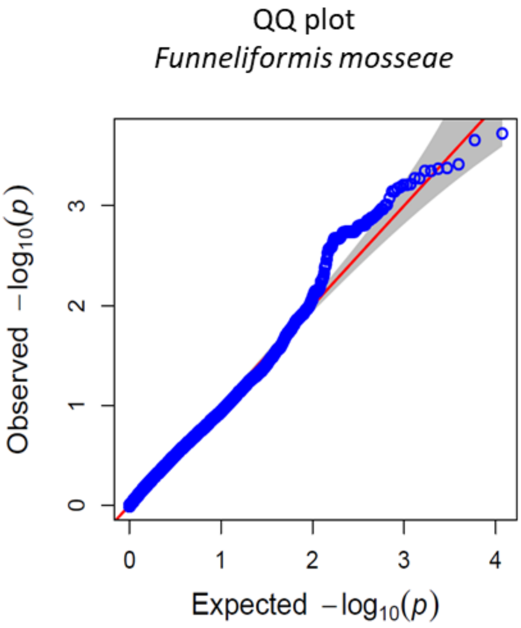
QQ plot
Funneliformis mosseae
Observed −log10(p)
Expected −log10(p)
0 1 2 3 4

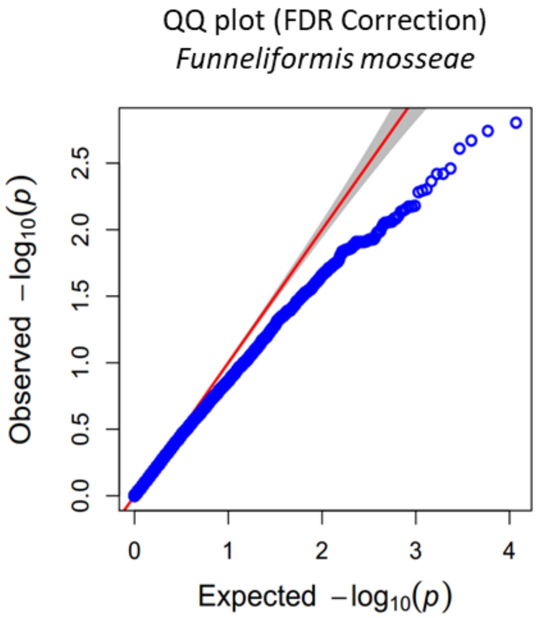
QQ plot (FDR Correction)
Funneliformis mosseae
Observed −log10(p)
Expected −log10(p)
0.0 0.5 1.0 1.5 2.0 2.5
0 1 2 3 4

Supplementary Information

Table S1 List of accessions of *Triticum turgidum* subspecies included in Exp. 1 and Exp. 2

Code	Name	Exp.1	Exp. 2
Triticum turgidum L. ssp. *durum* (Desf.) Husn.			
AG-1	5-BIL42	x	x
AG-3	Adamello		x
AG-4	Agridur		x
AG-5	Alemanno		x
AG-6	Altar84		x
AG-7	Ambral		x
AG-8	Ancomarzio		x
AG-9	Antas		x
AG-10	Appio	x	x
AG-11	Appulo	x	x
AG-12	Arcangelo	x	x
AG-13	Arcobaleno		x
AG-14	Ares (=Ionio)		x
AG-16	Athena		x
AG-17	Aziziah	x	x
AG-19	Belfuggito	x	x
AG-20	Berillo	x	x
AG-21	Brindur		x
AG-22	Cannizzo	x	x
AG-24	Capeiti-8	x	x
AG-25	Cappelli	x	x
AG-26	Casanova	x	x
AG-27	Ceedur		x
AG-28	Chiara		x
AG-29	Ciccio	x	x
AG-31	Cirillo	x	x
AG-32	Claudio	x	x
AG-33	Colosseo	x	x
AG-35	Creso	x	x
AG-36	Fauno (=Dauno)		x
AG-37	Doral		x
AG-39	Duilio	x	x
AG-40	Durfort	x	x
AG-41	Dylan	x	x
AG-43	Exeldur		x
AG-44	Fauno	x	x
AG-45	Fenix	x	x
AG-47	Fortore	x	x
AG-48	Gianni	x	x
AG-49	Giotto		x
AG-50	Granizo		x
AG-51	Grazia	x	x
AG-52	Grecale	x	x
AG-53	Grifoni 235	x	x

AG-54	Hymera		x
AG-56	Iride	x	x
AG-57	Isa	x	x
AG-58	Italo	x	x
AG-59	K26		x
AG-60	Karel	x	x
AG-63	L092		x
AG-64	L252		x
AG-65	Lambro	x	x
AG-66	Langdon		x
AG-67	Latino	x	x
AG-69	Lloyd		x
AG-70	Maestrale	x	x
AG-71	Meridiano	x	x
AG-72	Messapia	x	x
AG-73	Mexicali 75		x
AG-74	Mida	x	x
AG-75	Nefer		x
AG-76	Neodur	x	x
AG-77	Neolatino	x	x
AG-78	Normanno	x	x
AG-79	Ofanto	x	x
AG-80	Orfeo		x
AG-81	Martino		x
AG-83	Parsifal	x	x
AG-84	Pedroso	x	x
AG-85	Platani	x	x
AG-86	Plinio		x
AG-87	Polesine	x	x
AG-88	PR22D89	x	x
AG-89	Preco	x	x
AG-90	Primadur	x	x
AG-91	Produra	x	x
AG-92	Provenzal		x
AG-93	Quadruro		x
AG-94	Quadrato		x
AG-95	Russello	x	x
AG-97	S99B34		x
AG-98	Saadi		x
AG-99	Amedeo		x
AG-100	San Carlo	x	x
AG-102	Sansone	x	x
AG-103	Saragolla	x	x
AG-105	Simeto	x	x
AG-107	Svevo	x	x
AG-108	Taganrog	x	x
AG-109	Timilia	x	x

AG-110	Tito	x	x
AG-111	Tiziana	x	x
AG-112	Tresor	x	x
AG-113	Trinakria	x	x
AG-114	UC1113	x	x
AG-115	Valforte	x	x
AG-116	Valgerardo	x	x
AG-117	Valnova	x	x
AG-118	Varano	x	x
AG-119	Vendetta		x
AG-120	Vesuvio		x
AG-121	Bronte		x
AG-122	Virgilio		x
AG-123	Vitromax		x
AG-124	West Bread 881		x
AG-125	Zenit	x	x
AG-166	Baio		x
AG-300	Biancuccia	x	
AG-301	Ciciredda	x	
AG-302	Realforte	x	
AG-303	Bufalanera	x	
AG-304	Maiorcone	x	
AG-306	Scorsonera	x	
Triticum turgidum L. ssp. *dicoccoides* (Körn. exAsch. &Graebner) Thell			
AG-223	MG4337/198	x	
AG-234	PI 352332	x	
AG-235	PI 467029	x	
AG-236	PI 470944	x	
AG-225	MG5444/235	x	
AG-218	MG4343	x	
AG-219	MG29896	x	
AG-232	PI 355459	x	
AG-224	MG4328/61	x	
Triticum turgidum L. ssp. *dicoccum* (Schrank ex Schübler) Thell.			
AG-198	MG3521	x	
AG-200	MG5473	x	
AG-212	Molise Colli	x	
AG-201	Farvento	x	
AG-199	MG5323	x	
AG-204	MG4387	x	
AG-203	MG5350	x	
AG-202	Lucanica	x	
AG-210	MG5293/1	x	
AG-211	MG29704	x	
AG-308	Farro Italia Centrale	x	
AG-307	FarroGarfagnana	x	
AG-309	FarroMolisano	x	

Supplementary Information

Table S2 List of the Marker Trait Associations (MTAs) identified with the SUPER model for each AM fungal specie.

**Exp 2 Rhiz. Irregulare**			
SNP	Chromosome	Position	P.value
SNP_32805	3B	148,4	8,52E-05
SNP_48327	3B	148,4	3,27E-04
SNP_34450	2B	181,6	3,36E-04
SNP_47123	1A	19,5	6,12E-04
SNP_21579	1A	19,6	6,40E-04
SNP_34779	1A	16,7	7,42E-04
SNP_08151	7B	138,5	1,02E-03
SNP_51029	1A	19,5	1,20E-03
SNP_19704	1A	19,5	1,20E-03
SNP_69805	1A	19,7	1,20E-03
SNP_21580	1A	19,5	1,20E-03
SNP_72127	1A	19,6	1,38E-03
SNP_07351	1A	19,6	1,38E-03
SNP_68393	1A	19,7	1,38E-03
SNP_35266	1A	19,5	1,40E-03
SNP_12126	1A	19,6	1,40E-03
SNP_72258	1A	19,6	1,40E-03
SNP_52990	1A	19,7	1,40E-03
SNP_39784	1A	20,2	1,43E-03
SNP_36117	1A	16,7	1,59E-03
SNP_01073	1A	20,2	1,72E-03
SNP_35677	1B	150,9	1,77E-03
SNP_03519	1A	20,2	1,87E-03
SNP_54205	1A	20,2	1,93E-03
SNP_72793	1A	19,7	1,94E-03
SNP_34652	1B	150,9	2,05E-03
SNP_05889	1A	19,8	2,06E-03
SNP_72794	1A	19,7	2,08E-03
SNP_12175	7B	138,5	2,11E-03
SNP_75603	7B	138,5	2,18E-03

SNP_07627	1A	19,6	2,29E-03
SNP_70138	1A	19,8	2,31E-03
SNP_67280	1A	20,2	2,46E-03
SNP_67281	1A	20,2	2,46E-03
SNP_36053	1A	19,8	2,47E-03
SNP_66235	6A	85,4	2,48E-03
SNP_67282	1A	20,2	2,49E-03
SNP_75604	7B	138,5	2,54E-03
SNP_66667	7B	138,5	2,68E-03
SNP_75558	7B	138,5	3,04E-03
SNP_20174	2B	183,1	3,04E-03
SNP_74541	1A	19,8	3,14E-03
SNP_63578	3A	50,6	3,34E-03
SNP_71480	3A	50,6	3,34E-03
SNP_34302	7B	138,5	3,43E-03
SNP_73640	3A	48,4	3,61E-03
SNP_71478	3A	50,6	3,62E-03
SNP_05896	1A	19,8	3,71E-03
SNP_71479	3A	50,6	3,74E-03
SNP_07233	1A	20,2	3,77E-03
SNP_75800	6A	85,4	3,84E-03
SNP_11206	6A	85,7	3,95E-03
SNP_59719	3A	50,5	4,07E-03
SNP_73476	4A	22,2	4,23E-03
SNP_71474	7B	139,2	4,26E-03
SNP_80762	6A	85,7	4,29E-03
SNP_81476	6A	85,4	4,31E-03
SNP_72440	6A	85,7	4,42E-03
SNP_71477	3A	50,6	4,46E-03
SNP_76295	1A	16,5	4,55E-03
SNP_71473	7B	139,2	4,73E-03
SNP_80895	6A	85,7	4,79E-03
SNP_71171	1A	16,5	4,91E-03

SNP_78757	3A	48,4	5,03E-03
SNP_06837	3A	48,4	5,03E-03
SNP_68232	2B	174,6	5,06E-03
SNP_50029	1A	16,5	5,07E-03
SNP_76143	6A	21,4	5,11E-03
SNP_73272	1A	19,2	5,29E-03
SNP_04592	2B	180,1	5,31E-03
SNP_07569	2B	181,6	5,50E-03
SNP_14135	2B	181,6	5,69E-03
SNP_72128	1A	19,6	5,76E-03
SNP_56884	5B	48,9	5,78E-03
SNP_73615	1A	18,4	5,87E-03
SNP_71837	5A	0,1	5,92E-03
SNP_12428	5B	192,7	5,97E-03
SNP_31746	1A	19,8	6,07E-03
SNP_25037	2B	185,8	6,18E-03
SNP_78576	5A	0,7	6,28E-03
SNP_05563	6A	0,1	6,38E-03
SNP_41814	7B	139,2	6,70E-03
SNP_64321	5A	0,7	6,81E-03
SNP_72219	6A	85,7	7,16E-03
SNP_50962	7A	15,8	7,20E-03
SNP_72531	1A	10,6	7,24E-03
SNP_23361	7B	195,3	7,31E-03
SNP_73137	6A	85,7	7,59E-03
SNP_60121	5A	0	7,60E-03
SNP_50651	4B	135,5	7,90E-03
SNP_33471	5A	0,1	8,01E-03
SNP_74736	2B	185,7	8,36E-03
SNP_00389	1A	16,7	8,38E-03
SNP_19101	1A	19,5	8,41E-03
SNP_69933	6A	85,4	8,61E-03
SNP_71687	1A	10,8	8,63E-03

SNP_00290	5A	0	9,48E-03
SNP_71688	1A	10,8	9,64E-03
SNP_19563	2A	188,5	9,81E-03
SNP_52947	2B	156,6	9,84E-03
SNP_69630	2B	156,6	9,84E-03
SNP_81486	2B	156,6	9,84E-03
Exp 2 Fun. mosseae			
SNP	Chromosome	Position	P.value
SNP_63429	3A	77,4	1,89E-04
SNP_63430	3A	77,4	2,17E-04
SNP_67306	2A	46,6	3,82E-04
SNP_52139	2A	46,6	4,18E-04
SNP_63431	3A	77,4	4,25E-04
SNP_62348	2A	47,2	4,42E-04
SNP_67307	2A	46,6	4,45E-04
SNP_72621	3A	79,3	5,25E-04
SNP_30678	3A	110,2	5,26E-04
SNP_49517	2A	47,2	6,07E-04
SNP_43469	2A	46,6	6,10E-04
SNP_70098	2A	46,6	6,10E-04
SNP_70473	2A	47,2	6,50E-04
SNP_67304	2A	46,6	6,72E-04
SNP_67303	2A	46,6	7,11E-04
SNP_67305	2A	46,6	7,11E-04
SNP_22715	3A	110,2	8,36E-04
SNP_56835	3A	79,5	9,64E-04
SNP_78290	3A	80,3	9,82E-04
SNP_78928	3A	79,5	1,07E-03
SNP_77272	3A	79,4	1,07E-03
SNP_28467	3A	110,2	1,12E-03
SNP_05680	3A	77,4	1,19E-03
SNP_30130	3A	79,5	1,23E-03

SNP_55093	3A	79,5	1,26E-03
SNP_75077	3A	79,5	1,31E-03
SNP_80820	3A	79,5	1,31E-03
SNP_72494	7A	143,2	1,39E-03
SNP_30131	3A	79,5	1,39E-03
SNP_77040	2A	46,6	1,41E-03
SNP_78950	3A	79,5	1,52E-03
SNP_79869	3A	79,5	1,55E-03
SNP_72075	3A	79,5	1,55E-03
SNP_09982	3A	80,2	1,55E-03
SNP_69595	3A	79,5	1,56E-03
SNP_65513	3A	80,2	1,59E-03
SNP_72653	3A	80,3	1,59E-03
SNP_60910	3A	79,3	1,60E-03
SNP_72074	3A	93,4	1,73E-03
SNP_14015	3A	79,5	1,75E-03
SNP_00189	3A	79,4	1,81E-03
SNP_79510	3A	79,5	1,81E-03
SNP_36660	3A	79,5	1,81E-03
SNP_76006	3A	79,5	1,81E-03
SNP_81243	3A	79,5	1,81E-03
SNP_80728	3A	79,5	1,81E-03
SNP_49571	3A	79,3	1,81E-03
SNP_35463	3A	79,5	1,81E-03
SNP_47837	3A	79,5	1,81E-03
SNP_76478	3A	79,5	1,81E-03
SNP_80293	3A	79,5	1,81E-03
SNP_03210	3A	79,5	1,81E-03
SNP_77877	3A	110,2	1,83E-03
SNP_77875	3A	110,2	1,83E-03
SNP_79483	3A	110,2	1,83E-03
SNP_72591	3A	77,1	1,92E-03
SNP_72560	3A	79,5	1,98E-03

SNP_60421	3A	79,5	2,04E-03
SNP_23156	3A	80,3	2,06E-03
SNP_72686	3A	79,4	2,10E-03
SNP_65564	3A	79,5	2,10E-03
SNP_65798	3A	79,5	2,10E-03
SNP_70313	3A	79,5	2,10E-03
SNP_73850	3A	79,4	2,10E-03
SNP_03645	3A	79,5	2,10E-03
SNP_47885	3A	79,5	2,10E-03
SNP_78102	3A	79,5	2,10E-03
SNP_72652	3A	80,3	2,10E-03
SNP_07614	3A	79,5	2,13E-03
SNP_72687	3A	79,4	2,24E-03
SNP_78177	3A	79,5	2,35E-03
SNP_49202	3A	78	2,54E-03
SNP_69157	3A	77,8	2,58E-03
SNP_73130	3A	77,8	2,58E-03
SNP_61572	3A	77,8	2,58E-03
SNP_58095	3A	79,5	2,65E-03
SNP_70165	3A	81,1	2,68E-03
SNP_73976	4A	22,2	2,68E-03
SNP_06860	4A	25,2	2,73E-03
SNP_01403	1A	112,1	2,96E-03
SNP_06774	3A	80,9	2,96E-03
SNP_76143	6A	21,4	3,38E-03
SNP_02704	3A	79,5	3,44E-03
SNP_54775	3A	80,5	3,79E-03
SNP_11606	4A	25,2	4,03E-03
SNP_70442	4A	25,2	4,03E-03
SNP_04121	3B	42,5	4,14E-03
SNP_70443	4A	25,2	4,66E-03
SNP_72139	3A	81,1	5,01E-03
SNP_73371	1B	21,6	5,14E-03

SNP_72035	3A	86,9	5,28E-03
SNP_56681	6A	21,4	5,50E-03
SNP_75497	3A	80,9	5,57E-03
SNP_10508	3A	80,9	5,67E-03
SNP_33677	3A	80,9	5,67E-03
SNP_68422	3A	74,1	5,72E-03
SNP_28493	1A	112,2	6,31E-03
SNP_74657	7B	112,5	6,50E-03
SNP_34093	1B	19,4	6,68E-03
SNP_72195	3B	209,6	6,72E-03
SNP_81494	3A	80,9	6,88E-03
SNP_06100	1B	23,2	7,01E-03
SNP_33676	3A	80,9	7,13E-03
SNP_71639	3A	80,9	7,13E-03
SNP_73647	3A	80,9	7,13E-03
SNP_67048	3A	80,8	7,13E-03
SNP_66448	3A	80,9	7,13E-03
SNP_07896	3A	80,9	7,13E-03
SNP_72102	3A	80,9	7,13E-03
SNP_67654	3A	78	7,15E-03
SNP_35997	5A	213,8	7,15E-03
SNP_42993	1A	16,3	7,31E-03
SNP_37873	2B	148	7,38E-03
SNP_36797	1A	16,4	7,58E-03
SNP_24311	3B	41,3	7,84E-03
SNP_40846	5A	54,6	8,00E-03
SNP_09012	3A	80,9	8,05E-03
SNP_63934	3A	184	8,63E-03
SNP_60878	2B	75,4	8,67E-03
SNP_12829	5A	57,7	8,70E-03
SNP_72656	3B	209,6	8,75E-03
SNP_72193	3B	209,6	8,92E-03
SNP_72194	3B	209,6	9,33E-03

SNP_08435	3B	41,3	9,45E-03
SNP_19201	3B	8,8	9,46E-03
SNP_32801	3A	80,9	9,49E-03
SNP_74967	3A	78,8	9,90E-03

Supplementary Information

Table S3 Candidate genes identified for all the sequences of the markers mapped in the regions of the MTAs.

NameQTL	Chr.	Peak marker	Confidence Interval (±3cM)	cM	BLAST for Wild Emmer Genome Assembly (Zavitan WEWSeq v.1.0)
QTamf-1A	1A		IAAV3809	16,7	ERD (early-responsive to dehydration stress) family protein
			RAC875_c23587_271	16,7	ERD (early-responsive to dehydration stress) family protein
			wsnp_Ex_c7252_12452995	16,7	pumilio 3
			wsnp_Ex_c7252_12453079	16,7	pumilio 3
			BS00081680_51	16,7	ERD (early-responsive to dehydration stress) family protein
			BS00081682_51	16,7	ERD (early-responsive to dehydration stress) family protein
			BobWhite_c27474_124	16,7	General transcription factor IIH subunit 5
			BobWhite_c27474_65	16,7	General transcription factor IIH subunit 5
			Excalibur_c10065_570	16,7	Disease resistance protein
			Excalibur_c22240_938	16,7	Disease resistance protein (CC-NBS-LRR class) family
			Excalibur_c42185_156	16,7	unknown protein
			Excalibur_c45969_370	16,7	Disease resistance protein
			IACX6155	16,7	ERD (early-responsive to dehydration stress) family protein
			IACX9458	16,7	undescribed protein
			BobWhite_c1265_247	16,7	ERD (early-responsive to dehydration stress) family protein
			BobWhite_c685_1231	16,7	Disease resistance protein
			Kukri_c23081_2360	16,7	unknown protein
			Kukri_c2464_592	16,7	Disease resistance protein
			Kukri_c37592_531	16,7	unknown function
			RAC875_c1852_709	16,7	Disease resistance protein
			RAC875_c53568_391	16,7	unknown function
			RAC875_c5485_139	16,7	pumilio 3
			tplb0043h23_1346	16,7	Elongation factor 2
			Tdurum_contig83113_134	18,4	undescribed protein
			wsnp_BE438866A_Ta_2_8-0_T_R_1891196599	18,4	NAD(P)-binding Rossmann-fold superfamily protein
			Excalibur_c105151_200	19,5	ATP-dependent RNA helicase DBP7
			IAAV6899	19,5	ATP-dependent RNA helicase DBP7
			Kukri_rep_c108031_378	19,5	ATP-dependent RNA helicase DBP7
			RAC875_c108229_54	19,5	ATP-dependent RNA helicase DBP7
			D_GDEEGVY02H7D8K_299	19,5	protein kinase family protein

			Excalibur_c20777_428	19,5	FAR1-related sequence 5
			BS00026453_51	19,6	receptor kinase 2
			CAP7_c1645_359	19,6	Protein NRT1/ PTR FAMILY 6.2
			Excalibur_c20777_315	19,6	FAR1-related sequence 5
			Tdurum_contig15335_452	19,7	Glycosyltransferase family 61 protein
			RAC875_c2122_159	19,7	mRNA export factor
			Tdurum_contig61492_458	19,7	F-box/RNI-like superfamily protein
		SNP_72794	Tdurum_contig61492_684	19,7	F-box/RNI-like superfamily protein
			BS00003761_51	19,8	Outer envelope pore protein 16, chloroplastic
			BS00003802_51	19,8	undescribed protein
			IACX5892	19,8	Outer envelope pore protein 16, chloroplastic
			BS00082266_51	19,8	undescribed protein
			RAC875_c54245_88	19,8	F-box/RNI-like superfamily protein
			IAAV1469	20,1	embryo defective 2735
			BS00012052_51	20,3	mRNA export factor
			BobWhite_c1890_712	20,3	mRNA export factor
			CAP8_c1356_189	20,3	Protein NRT1/ PTR FAMILY 6.2
			tplb0036l23_696	20,3	Protein NRT1/ PTR FAMILY 6.2
			wsnp_Ex_c64327_63176640	20,7	Poly [ADP-ribose] polymerase 1
			Kukri_rep_c72397_600	20,7	Poly [ADP-ribose] polymerase 1
			RAC875_c2654_1817	20,7	Poly [ADP-ribose] polymerase 1
			RAC875_c37934_285	20,7	actin depolymerizing factor 4
			RAC875_c56994_301	20,7	Poly [ADP-ribose] polymerase 1
			BS00107852_51	20,7	BRI1-KD interacting protein 130
			Excalibur_c110483_103	20,7	Poly [ADP-ribose] polymerase 1
			Excalibur_c81545_101	20,7	undescribed protein
			Ku_c20069_531	20,7	UDP-D-apiose/UDP-D-xylose synthase 2
			Kukri_c24144_1568	20,7	Poly [ADP-ribose] polymerase 1
			RAC875_c13012_1598	20,7	RNA-binding protein 39
			RAC875_c37934_225	20,7	actin depolymerizing factor 4
			BS00039749_51	20,7	Pathogenesis-related thaumatin superfamily protein
			Excalibur_c18694_273	20,9	RNA-binding protein 39
			Excalibur_c22642_90	20,9	RNA-binding protein 39
			Excalibur_rep_c92985_510	20,9	Glucose-6-phosphate isomerase
QTamf-2B	2B		RAC875_c4602_445	178,5	Cysteine protease 1
			IACX6292	180,1	beta glucosidase 13
			Kukri_c21135_1071	180,1	U4/U6.U5 tri-snRNP-associated protein 1

			RAC875_rep_c119322_54	180,1	beta glucosidase 13
			TA002989-0535	180,1	beta glucosidase 13
			wsnp_Ex_rep_c103381_88353000	180,1	beta glucosidase 13
			IACX8602	180,1	Cysteine protease 1
			BobWhite_c928_311	180,1	RNA pyrophosphohydrolase
			RAC875_c4602_886	180,1	Cysteine protease 1
			Tdurum_contig28437_135	180,1	Cysteine protease 1
			BS00083329_51	180,1	undescribed protein
			Excalibur_c23857_594	180,1	Choline transporter-like protein 2
			Excalibur_c58935_208	180,1	phosphomannomutase
			BobWhite_c12911_649	180,1	Acyl-CoA-binding domain-containing protein 1
			RAC875_rep_c71149_738	180,1	beta glucosidase 13
			Tdurum_contig82379_629	180,1	beta glucosidase 13
			Kukri_c94792_127	181,1	pentatricopeptide repeat 336
			BobWhite_c20158_834	181,6	Protein kinase superfamily protein
			CAP7_c4827_53	181,6	4-coumarate:CoA ligase 2
			CAP7_c4827_78	181,6	4-coumarate:CoA ligase 2
			Excalibur_c12675_1147	181,6	K(+) efflux antiporter 2, chloroplastic
			Excalibur_c2993_457	181,6	C2 calcium/lipid-binding plant phosphoribosyltransferase family
			Excalibur_rep_c67599_677	181,6	C2 calcium/lipid-binding plant phosphoribosyltransferase family
			Jagger_c10188_98	181,6	Protein kinase family protein
			Kukri_c17_967	181,6	Protein kinase superfamily protein
			Kukri_c60352_344	181,6	C2 calcium/lipid-binding plant phosphoribosyltransferase family
			Tdurum_contig27848_179	181,6	AT-hook motif nuclear-localized protein 1
			CAP7_c2746_331	181,6	AT-hook motif nuclear-localized protein 1
		SNP_34450	IAAV1798	181,6	K(+) efflux antiporter 2, chloroplastic
			IAAV5401	181,6	C2 calcium/lipid-binding plant phosphoribosyltransferase family
			Kukri_c17_1246	181,6	Protein kinase superfamily protein
			wsnp_Ex_c31064_39902843	181,6	Protein kinase superfamily protein
			wsnp_Ex_c41300_48154348	181,6	C2 calcium/lipid-binding plant phosphoribosyltransferase family
			wsnp_Ex_c5193_9204522	181,6	C2 calcium/lipid-binding plant phosphoribosyltransferase family
			BS00109546_51	181,6	Cytochrome P450 superfamily protein
			D_contig64884_675	181,6	Inorganic pyrophosphatase

			Kukri_c16249_92	181,6	beta glucosidase 13
			RAC875_c31214_58	181,6	beta glucosidase 13
			RAC875_rep_c71149_1446	181,6	beta glucosidase 13
			BS00072839_51	181,6	Glutathione S-transferase family protein
			BS00072840_51	181,6	Glutathione S-transferase family protein
			Excalibur_c5193_64	181,6	C2 calcium/lipid-binding plant phosphoribosyltransferase family
			Excalibur_c73791_215	181,6	Glutathione S-transferase family protein
			Excalibur_c77068_166	181,6	Glutathione S-transferase family protein
			Excalibur_c8919_345	181,6	Regulator of chromosome condensation (RCC1) family protein
			Kukri_c3501_1175	181,6	Regulator of chromosome condensation (RCC1) family protein
			RAC875_c10626_2089	181,6	K(+) efflux antiporter 2, chloroplastic
			BS00007384_51	181,6	Cytochrome P450 superfamily protein
			RAC875_c999_612	181,6	Disease resistance protein
			RFL_Contig4583_3169	181,6	Regulator of chromosome condensation (RCC1) family protein
			BS00100117_51	182,7	Cleavage and polyadenylation specificity factor subunit 5
			Excalibur_c12675_1395	182,7	K(+) efflux antiporter 2, chloroplastic
			wsnp_Ex_c12675_20144479	182,7	K(+) efflux antiporter 2, chloroplastic
			IAAV5743	182,7	Cleavage and polyadenylation specificity factor subunit 5
			RAC875_c63343_52	182,7	Cleavage and polyadenylation specificity factor subunit 5
			CK162551	182,7	EamA-like transporter family protein
			wsnp_Ex_rep_c70525_69448648	182,7	transportin 1
			wsnp_JD_c30_49073	182,7	phosphatidylinositol-4-phosphate 5-kinase family protein
			wsnp_Ku_c28820_38731137	182,7	transportin 1
			BobWhite_c18071_171	182,7	unknown function
			BobWhite_c18406_110	182,7	tubulin folding cofactor B
			CAP12_c3254_366	182,7	Vacuolar iron transporter homolog 5
			CAP12_c449_277	182,7	oligopeptide transporter 4
			BobWhite_c1105_745	182,7	oligopeptide transporter 4
			D_contig63990_376	182,7	receptor-like protein kinase 1
			Excalibur_c2311_1021	182,7	DDB1- and CUL4-associated factor homolog 1
			Excalibur_c2311_114	182,7	DDB1- and CUL4-associated factor homolog 1

			Excalibur_c2311_1563	182,7	DDB1- and CUL4-associated factor homolog 1
			Excalibur_c25043_1020	182,7	transportin 1
			Excalibur_c25043_1221	182,7	transportin 1
			Excalibur_c25043_357	182,7	transportin 1
			Excalibur_c3635_3192	182,7	Acyl-CoA N-acyltransferase with RING/FYVE/PHD-type zinc finger protein
			Excalibur_c6937_1065	182,7	DNA-directed RNA polymerase II subunit 1
			Excalibur_c7366_1926	182,7	DDB1- and CUL4-associated factor homolog 1
			Excalibur_rep_c105614_172	182,7	Acyl-CoA N-acyltransferase with RING/FYVE/PHD-type zinc finger protein
			GENE-0676_715	182,7	DDB1- and CUL4-associated factor homolog 1
			IACX7717	182,7	mevalonate kinase
			Kukri_c16035_685	182,7	DDB1- and CUL4-associated factor homolog 1
			Kukri_c20793_249	182,7	Protein MCM10 homolog
			Kukri_c39136_675	182,7	1-(5-phosphoribosyl)-5-[(5-phosphoribosylamino)methylideneamino] imidazole-4-carboxamide isomerase, chloroplastic
			Kukri_c49784_56	182,7	Zinc finger protein ZPR1 homolog
			Kukri_c49784_86	182,7	Zinc finger protein ZPR1 homolog
			Kukri_c8494_77	182,7	Acyl-CoA N-acyltransferase with RING/FYVE/PHD-type zinc finger protein
			Kukri_c900_1334	182,7	Acyl-CoA N-acyltransferase with RING/FYVE/PHD-type zinc finger protein
			Kukri_c900_1376	182,7	Acyl-CoA N-acyltransferase with RING/FYVE/PHD-type zinc finger protein
			Kukri_c9898_1766	182,7	CsAtPR5
			Kukri_rep_c101484_438	182,7	DNA-directed RNA polymerase II subunit 1
			RAC875_c16993_196	182,7	Protein kinase family protein
			RAC875_c18928_455	182,7	nodulin MtN21 /EamA-like transporter family protein
			RAC875_c18928_529	182,7	nodulin MtN21 /EamA-like transporter family protein
			RAC875_c19042_2102	182,7	Acyl-CoA N-acyltransferase with RING/FYVE/PHD-type zinc finger protein
			RAC875_c19042_443	182,7	Acyl-CoA N-acyltransferase with RING/FYVE/PHD-type zinc finger protein
			RAC875_c3166_379	182,7	Small glutamine-rich tetratricopeptide repeat-containing protein
			RAC875_c3259_276	182,7	DDB1- and CUL4-associated factor homolog 1

			BS00004413_51	182,7	RNA pyrophosphohydrolase
			RAC875_rep_c111384_441	182,7	beta glucosidase 13
			RAC875_rep_c71149_148	182,7	beta glucosidase 13
			BS00025538_51	182,7	unknown function
			BS00031097_51	182,7	RING/U-box superfamily protein
			BS00039185_51	182,7	Calcium-dependent lipid-binding (CaLB domain) family protein
			wsnp_CAP11_c269_233382	183,1	Thioredoxin superfamily protein
			Excalibur_c94383_157	183,1	Regulator of chromosome condensation (RCC1) family protein
			tplb0053o16_915	183,1	Inorganic pyrophosphatase
			wsnp_Ra_c14711_22960624	183,1	Protein kinase family protein
			Excalibur_c12675_1789	183,1	K(+) efflux antiporter 2, chloroplastic
			Jagger_c7527_89	183,1	isopropylmalate dehydrogenase 2
			RFL_Contig5277_714	183,1	isopropylmalate dehydrogenase 2
			tplb0053o16_838	183,1	Inorganic pyrophosphatase
			wsnp_Ex_rep_c67697_66363222	183,1	C2 calcium/lipid-binding plant phosphoribosyltransferase family
			Kukri_c52900_280	183,1	unknown function
			RAC875_c29056_405	183,1	Prenylcysteine oxidase
			Tdurum_contig20987_1271	183,1	undescribed protein
			BS00083998_51	183,6	30S ribosomal protein S11
			RFL_Contig5495_563	183,6	30S ribosomal protein S11
			wsnp_Ex_c16074_24502385	183,6	Peroxidase superfamily protein
			RAC875_c17798_66	183,6	Peroxidase superfamily protein
			BS00106597_51	183,6	Peroxidase superfamily protein
			BS00106606_51	183,6	unknown function
			CJ577969	183,7	Cleavage and polyadenylation specificity factor subunit 5
QTamf-5A	5A		RAC875_rep_c114788_238	0,0	unknown function
			Tdurum_contig47762_975	0,0	sucrose transporter 4
			Tdurum_contig47762_1254	0,0	sucrose transporter 4
			Excalibur_c63045_203	0,0	sucrose transporter 4
			SBG_117188	0,1	sucrose transporter 4
			Tdurum_contig47762_1140	0,1	sucrose transporter 4
			GENE-3572_70	0,1	sucrose transporter 4
		SNP_29990	Ex_c14898_407	0,1	unknown function
			RFL_Contig3370_343	0,7	expansin B2

			wsnp_Ex_c6209_10838456	0,7	carotenoid cleavage dioxygenase 1
			TA004832-0873	0,7	carotenoid cleavage dioxygenase 1
			wsnp_Ex_c3620_6612231	1,7	sucrose-phosphatase 1
			BobWhite_rep_c67379_241	1,7	P-loop containing nucleoside triphosphate hydrolases superfamily
			wsnp_JD_c2128_2930150	1,7	sucrose-phosphatase 1
			wsnp_Ex_c3620_6612294	1,7	sucrose-phosphatase 1
			BS00099534_51	2,8	HIPL1 protein
			SBG_187955	3,0	Pentatricopeptide repeat-containing protein
			RAC875_c2061_55	3,0	Plasma membrane ATPase 3
			Excalibur_c22411_690	3,0	Plasma membrane ATPase 3
			RAC875_c2128_1207	3,0	undescribed protein
			RAC875_c2061_292	3,0	Plasma membrane ATPase 3
QTamf-6A.1	6A		Tdurum_contig50062_934	16,7	Acylamino-acid-releasing enzyme
			RAC875_rep_c106371_205	16,7	Acylamino-acid-releasing enzyme
			1147033|10880520	21,0	glutamate receptor 3.3
			SBG_166068	21,4	RNAligase isoform 2
			BobWhite_c32377_62	21,4	Protein kinase superfamily protein
			BobWhite_c32377_278	21,4	Protein kinase superfamily protein
			GENE-4011_673	21,4	Protein kinase superfamily protein
			CA716967	21,4	undescribed protein
			wsnp_Ex_c1050_2009301	21,4	undescribed protein
			RFL_Contig2954_581	21,4	GMP synthase [glutamine-hydrolyzing]
		SNP_76143	wsnp_Ex_c1011_1931956	21,4	60S ribosomal protein L13-1
			Tdurum_contig11414_310	21,4	glutamate receptor 3.3
			RFL_Contig1877_207	21,4	Alpha/beta-gliadin A-V
			Tdurum_contig15699_236	21,4	Peptidyl-prolyl cis-trans isomerase Pin1
			BobWhite_c32377_62	22,5	Protein kinase superfamily protein
			BobWhite_c32377_278	23,2	Protein kinase superfamily protein
			Tdurum_contig57699_344	24,3	Protein kinase superfamily protein
			Tdurum_contig57699_110	24,3	Protein kinase superfamily protein
QTamf-6A.2	6A		Tdurum_contig30251_210	94,5	undescribed protein
			BobWhite_c26503_61	95,0	undescribed protein
			RFL_Contig5314_1147	95,0	ankyrin repeat family protein
			Ku_c604_705	95,5	Autophagy-related protein 18
			Ku_c87838_1055	95,9	Isopentenyl-diphosphate Delta-isomerase

			RAC875_c11289_71	96,2	Vacuolar protein-sorting-associated protein 33 homolog
			Ex_c5868_1113	96,2	Vacuolar protein-sorting-associated protein 33 homolog
			Kukri_c19878_4002	96,5	Clustered mitochondria protein
			wsnp_Ex_rep_c68010_66754534	96,5	Clustered mitochondria protein
			wsnp_Ex_rep_c68010_66754801	96,5	Clustered mitochondria protein
			wsnp_Ex_rep_c68010_66754171	96,5	Clustered mitochondria protein
		SNP_76302	wsnp_Ex_c11439_18459047	97,4	no hits found
			Excalibur_c33709_68	97,6	Formin Homology 14
			Tdurum_contig27888_760	97,6	Serine incorporator 3
			BS00074024_51	97,6	purple acid phosphatase 10
QTamf-7A	7A		Tdurum_contig22364_523	136,4	Disease resistance protein
			Tdurum_contig62357_527	136,4	Core-2/I-branching beta-1,6-N-acetylglucosaminyltransferase family
			Kukri_rep_c105999_572	136,4	Core-2/I-branching beta-1,6-N-acetylglucosaminyltransferase family
			RAC875_rep_c111784_231	136,9	Copper-transporting ATPase 1
			wsnp_Ex_c5448_9619922-0_T_F_1891208110	137,1	Serrate RNA effector molecule
			wsnp_Ku_c34643_43968242	137,2	Zinc finger CCHC domain-containing protein 10
			Jagger_c8331_69	137,2	undescribed protein
			Kukri_c100378_51	137,2	unknown function
			wPt-6842	137,2	YELLOW STRIPE like 2
			BS00109319_51	137,5	exocyst subunit exo70 family protein G1
			wsnp_CAP7_c949_486485	137,5	undescribed protein
			Excalibur_c12996_775	137,5	exocyst subunit exo70 family protein G1
			wsnp_CAP11_c827_513472	137,8	Protein of unknown function (DUF1336)
			Tdurum_contig23024_763	137,8	receptor-like protein kinase 2
			RAC875_c57656_170	138,3	sulfotransferase 2A
			Tdurum_contig93258_281	138,7	xyloglucan endotransglucosylase/hydrolase 25
			Excalibur_c20486_268	138,7	undescribed protein
			Excalibur_c20307_654	138,7	undescribed protein
			wsnp_Ra_c22201_31650505	139,4	Plant protein of unknown function (DUF863)
			wsnp_Ex_c8823_14754501	139,4	Plant protein of unknown function (DUF863)

			Tdurum_contig27847_579	139,4	Xyloglucan endotransglucosylase/hydrolase family protein
			wsnp_Ra_c22201_31650648	139,4	Plant protein of unknown function (DUF863)
			RAC875_c22201_461	139,4	Plant protein of unknown function (DUF863)
			Ku_c2990_1997	139,4	undescribed protein
		SNP_75676	wsnp_BF482403B_Ta_1_1	139,4	unknown function
			IACX5974	139,4	undescribed protein
			RAC875_c60161_145	139,4	RING/U-box superfamily protein
			wsnp_Ku_rep_c113718_96236830	140,0	Protein of unknown function (DUF1644)
			Kukri_c41297_284	140,7	undescribed protein
			RAC875_c478_1364	142,5	Copper-transporting ATPase 1
			Tdurum_contig32378_366	142,5	undescribed protein
			RAC875_c47130_120	142,5	unknown function
QTamf-7B	7B		Kukri_c1957_581	109,2	Protein of unknown function (DUF1644)
			Tdurum_contig76289_1530	109,2	Protein of unknown function (DUF1644)
			Kukri_c1957_920	109,2	Protein of unknown function (DUF1644)
			Excalibur_c63900_370	109,2	Protein of unknown function (DUF1644)
			BS00105558_51	109,2	Protein of unknown function (DUF1644)
			Kukri_c58340_181	109,2	unknown function
			RAC875_c37125_308	111,0	F-box family protein
			Kukri_c91303_322	111,6	undescribed protein
			BobWhite_c27679_112	112,2	calmodulin 6
			RFL_Contig3869_808	112,2	Leucine-rich receptor-like protein kinase family protein
			Tdurum_contig34357_160	112,2	cytochrome B5-like protein
			wsnp_BF482403B_Ta_1_1	112,5	unknown function
			Tdurum_contig32378_439	112,5	undescribed protein
			tplb0039c07_334	112,5	Protein of unknown function (DUF679)
		SNP_35797	IACX1805	112,5	no hits found
			BobWhite_c5961_849	112,5	Heavy metal transport/detoxification superfamily protein
			RAC875_c570_302	112,5	cytochrome B5-like protein
			tplb0055p22_1895	112,5	Copper-transporting ATPase 1
			Tdurum_contig16482_124	113,4	Disease resistance protein
			Excalibur_c2217_68	113,4	2-oxoglutarate (2OG) and Fe(II)-dependent oxygenase superfamily
			RAC875_rep_c73821_223	113,4	2-oxoglutarate (2OG) and Fe(II)-dependent oxygenase superfamily

			Tdurum_contig43966_1107	113,4	2-oxoglutarate (2OG) and Fe(II)-dependent oxygenase superfamily
			Kukri_c38676_251	113,4	Disease resistance protein
			Tdurum_contig16482_252	113,4	Disease resistance protein
			Tdurum_contig43966_813	113,4	2-oxoglutarate (2OG) and Fe(II)-dependent oxygenase superfamily
			Tdurum_contig58110_441	113,4	Disease resistance protein
			wsnp_JD_c4813_5944683	113,4	RNA polymerase I specific transcription initiation factor RRN3
			BS00001144_51	113,4	RNA polymerase I specific transcription initiation factor RRN3
			wsnp_BE404339B_Ta_2_2	113,4	RNA polymerase I specific transcription initiation factor RRN3
			Tdurum_contig43966_1182	113,4	2-oxoglutarate (2OG) and Fe(II)-dependent oxygenase superfamily
			BobWhite_rep_c64772_309	113,4	RNA polymerase I specific transcription initiation factor RRN3
			wsnp_JD_c4813_5945085	113,4	RNA polymerase I specific transcription initiation factor RRN3
			RAC875_c5646_440	113,7	Galactosylgalactosylxylosylprotein 3-beta-glucuronosyltransferase 1
			Tdurum_contig28174_132	113,7	Galactosylgalactosylxylosylprotein 3-beta-glucuronosyltransferase 1
			Tdurum_contig28174_76	113,7	Galactosylgalactosylxylosylprotein 3-beta-glucuronosyltransferase 1
			Tdurum_contig29317_467	113,7	Galactosylgalactosylxylosylprotein 3-beta-glucuronosyltransferase 1
			RAC875_c37751_215	113,7	Galactosylgalactosylxylosylprotein 3-beta-glucuronosyltransferase 1
			RAC875_c5646_969	113,7	Galactosylgalactosylxylosylprotein 3-beta-glucuronosyltransferase 1
			Kukri_c4143_869	113,7	EH domain-containing protein 1
			RAC875_c18043_369	113,7	EH domain-containing protein 1
			BobWhite_c15497_73	113,7	Galactosylgalactosylxylosylprotein 3-beta-glucuronosyltransferase 1
			RAC875_c5646_774	113,7	undescribed protein
			RFL_Contig1404_351	113,7	unknown function
			Kukri_c4143_1055	113,7	EH domain-containing protein 1
			BobWhite_c23044_279	113,7	unknown function
			RAC875_c18043_411	113,7	EH domain-containing protein 1

			Kukri_c42156_327	113,7	unknown function
			Kukri_c8718_193	113,7	EH domain-containing protein 1
			Tdurum_contig3914_153	114,2	unknown function
			Excalibur_rep_c108075_472	114,2	unknown function
			RAC875_c6020_496	114,2	unknown function
			RAC875_c1742_2710	114,2	unknown function
			Excalibur_c6738_2072	114,2	unknown function
			Kukri_c17144_678	114,2	undescribed protein
			Tdurum_contig74753_946	114,9	Translation machinery associated TMA7
			Tdurum_contig98926_227	114,9	Translation machinery associated TMA7
			RAC875_c33333_266	114,9	Gag-Pol polyprotein
			IACX1302	114,9	auxin response factor 16
			Kukri_rep_c71173_2043	114,9	Ubiquitin carboxyl-terminal hydrolase 13
			RAC875_c7251_656	114,9	RAN binding protein 1
			RAC875_c17510_356	114,9	Gag-Pol polyprotein
			RAC875_c29004_652	114,9	undescribed protein
			Kukri_c42653_248	114,9	Calcium-dependent lipid-binding (CaLB domain) family protein
			Kukri_c42653_179	114,9	Calcium-dependent lipid-binding (CaLB domain) family protein
			wsnp_JD_c2701_3626787	114,9	Disease resistance protein RPM1
			Excalibur_c28715_447	114,9	Gag-Pol polyprotein
			BobWhite_c149_4419	114,9	unknown function
			BobWhite_c23044_279	114,9	unknown function
			RAC875_c64596_302	115,3	External alternative NAD(P)H-ubiquinone oxidoreductase B1, mitochondrial
			RAC875_c21795_966	115,3	External alternative NAD(P)H-ubiquinone oxidoreductase B1, mitochondrial
			GENE-4746_72	115,3	External alternative NAD(P)H-ubiquinone oxidoreductase B1, mitochondrial
			SBG_86356	115,7	2-oxoglutarate (2OG) and Fe(II)-dependent oxygenase superfamily protein
